# Layer-Specific Physiological Features and Interlaminar Interactions in the Primary Visual Cortex of the Mouse

**DOI:** 10.1016/j.neuron.2018.12.009

**Published:** 2019-02-06

**Authors:** Yuta Senzai, Antonio Fernandez-Ruiz, György Buzsáki

**Affiliations:** 1 Neuroscience Institute, New York University, Langone Medical Center, New York, NY 10016, USA; 2 Department of Neurology, Langone Medical Center, New York University, New York, NY 10016, USA; 3 Center for Neural Science, New York University, New York, NY 10003, USA

**Keywords:** primary visual cortex, laminar recordings, cortical layers, cell types, oscillations, current source density, alpha rhythm, sleep, optogenetics

## Abstract

The relationship between mesoscopic local field potentials (LFPs) and single-neuron firing in the multi-layered neocortex is poorly understood. Simultaneous recordings from all layers in the primary visual cortex (V1) of the behaving mouse revealed functionally defined layers in V1. The depth of maximum spike power and sink-source distributions of LFPs provided consistent laminar landmarks across animals. Coherence of gamma oscillations (30–100 Hz) and spike-LFP coupling identified six physiological layers and further sublayers. Firing rates, burstiness, and other electrophysiological features of neurons displayed unique layer and brain state dependence. Spike transmission strength from layer 2/3 cells to layer 5 pyramidal cells and interneurons was stronger during waking compared with non-REM sleep but stronger during non-REM sleep among deep-layer excitatory neurons. A subset of deep-layer neurons was active exclusively in the DOWN state of non-REM sleep. These results bridge mesoscopic LFPs and single-neuron interactions with laminar structure in V1.

## Highlights

• Multisite LFP recording and LFP-spike coupling identified physiological layers in V1
• The prominent 3–6 Hz LFP shared characteristic features with primate alpha rhythm
• Spike transmission strength from layer 2/3 to layer 5 neurons was stronger during waking
• A subset of layer 6 neurons was active selectively in the DOWN state of non-REM sleep

## Teaser

The relationship between LFP patterns and single-neuron firing in the visual cortex is identified by Senzai et al. by using high-density silicon probe recordings, ICA-based LFP analysis, LFP-spike coupling, and spike transmission probability in freely moving mice.

## Introduction

A characteristic feature of the neocortex is its laminar organization. The cortical columnar microcircuitry is viewed as a stack of interconnected yet distinct neuronal networks in which each lamina possesses somewhat unique patterns with different specific inputs, projection targets, and feedback connections (Mountcastle, 1997, Callaway, 1998, Douglas and Martin, 2004, Harris and Shepherd, 2015). How the laminar structure relates to mesoscopic physiological patterns, such as local field potential (LFP) oscillations and physiological interactions of single neurons across layers, is not well understood. Numerous oscillatory and transient LFP patterns of functional relevance have been described in various neocortical regions. However, their relationship to afferents, intracortical connectivity, and the firing patterns of individual neurons has been largely unexplored (Mitzdorf, 1987, Buzsáki et al., 2012).

Using multisite recording silicon probes that span all cortical layers, we sought to characterize layer-specific physiological patterns and neuronal cross-talk between layers in the primary visual cortex (V1) of freely behaving mice. The well-characterized anatomical connectivity and the diverse neuronal types of V1 (Jiang et al., 2015, Gouwens et al., 2018) make this cortical area ideal to investigate how the different cell types in different layers interact during physiological operations, such as sensory processing and offline states, such as sleep. Previous recordings in the hippocampus, using a similar approach, have led to a solid understanding of the relationship between extracellular signals and anatomical connectivity. LFP patterns can identify layers *in vivo* with <25–50 μm precision (Fujisawa et al., 2008, Berényi et al., 2014, Senzai and Buzsáki, 2017) and have been used to identify the unique relationship between upstream activity levels and hippocampal spike outputs and relate such input-output transformation to behavior (Fernández-Ruiz et al., 2017). Similar strategies have been followed in the V1 of waking monkeys (Schroeder et al., 1998, Maier et al., 2010, Xing et al., 2012, Dougherty et al., 2017) and other cortical areas in rodents, mostly under anesthesia (Sakata and Harris, 2012, Reyes-Puerta et al., 2015). In these previous studies, layer boundaries were estimated mainly by depth criteria, and the relationship among LFP depth profiles, neuronal activity, and interlayer interactions in different behavioral states was not addressed quantitatively.

Our goal was to define layers functionally in V1 of the mouse and relate them to classical anatomical layers. Because spontaneous mesoscopic patterns in the primate V1 are characterized by gamma (Engel et al., 2001, Atallah and Scanziani, 2009), alpha (Schroeder et al., 1998, Maier et al., 2010, Klimesch et al., 2007), and slow (Haider et al., 2013) oscillations, we searched for their corresponding patterns in the mouse. Finally, we examined the brain state-dependent spike transmission probabilities among putative principal cells and inhibitory interneurons across cortical layers in waking and sleep to quantify their brain state dependence.

## Results

### Anchoring Mesoscopic Patterns to Physiological Landmarks

**Figure 1:**
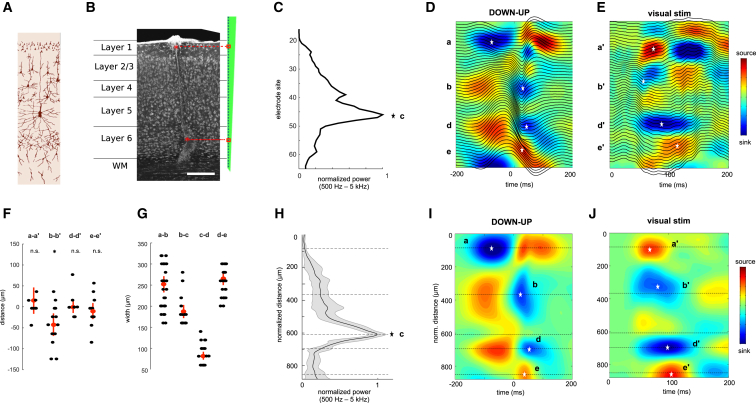
Electrophysiological Landmarks Identify V1 Layers (A) Schematic of the spatial arrangement of the main cellular types in V1. (B) Histological verification of probe location along V1 cellular layers. Small electrolytical lesions were performed with the two electrodes highlighted on the silicon probe sketch (red rectangles). (C) Multi-unit (MUA) spectral power (500 Hz to 5 kHz) distribution along probe track. (D and E) Average current source density (CSD) map and LFP traces for DOWN-UP transitions of non-REM sleep (D) and for (E) visual stimulation. (A)–(E) are from the same animal. Letters a–e and a′–e′ indicate the five electrophysiological landmarks chosen to align depth profiles across animals (asterisks). (F) Comparison of CSD landmarks from DOWN-UP transitions and visual evoked responses (n = 13 mice). Red dots and whiskers represent mean and SD; black dots individual animals. ^∗^p < 0.05 (signed rank test). (G) Distance variations between physiological landmarks across all animals. (H) Laminar distribution of MUA power (mean ± SEM; n = 19 mice) in normalized depth coordinates. (I and J) Average CSD depth profile of DOWN-UP transitions (n = 19 mice) (I) and (J) visual responses (n = 13 mice) in normalized depth coordinates.

Mice (n = 19) were implanted with a single-shank, 64-site linear silicon probe and recorded during free behavior in their home cage. The probe was placed parallel with the orientation of the apical dendrites of pyramidal neurons in the V1 (Figure 1). In our initial experiments, small electrolytic lesions were made to calibrate the positions of the recording sites with histological verification (n = 4; Figures 1A and S1). Subsequently, we used the characteristic depth distribution of LFPs and unit firing to identify five physiological landmarks that reliably anchored the recording sites in V1 across animals and recordings. The most prominent landmark was a large-amplitude peak of the depth profile of power between 500 Hz and 5 kHz corresponding to mid-layer 5 (Figure 1C, depth c). This peak likely reflected the aggregated power from the high firing rates of the large layer 5 pyramidal (Ray and Maunsell, 2010). The remaining four landmarks (Figures 1D and 1I, depths a, b, d, and e) were obtained from the depth distributions of the most prominent current source density (CSD) sinks and sources of spontaneous slow oscillations of non-rapid eye movement (REM) sleep (Steriade et al., 1993).

To investigate the origin of landmarks a–e, we compared the slow oscillation sinks and sources with those of light-evoked responses in the waking mouse (Figures 1E and 1J; 100 ms light pulses). Visual stimulation produced an early sink (b′) at the border between layers 3 and 4, which likely reflected afferent activation from the lateral geniculate body (Mitzdorf, 1987). The early sink was accompanied by a source in superficial layers (a′) and a sink-source pair corresponding to the border between layers 5 and 6 (sink, d′) and lower layer 6 (source, e′) (Reyes-Puerta et al., 2015, Niell and Stryker, 2008). Sink b, associated with the “UP” state of slow oscillation during non-REM sleep, was slightly but significantly deeper than the light-evoked sink b′ in the layer 3/4 border (39 ± 49 μm; p < 0.05, signed rank test; n = 13 mice; Figure 1F), whereas the other sinks and sources occurred at the same depth as those seen with visual stimulation (p > 0.05, signed rank test). Importantly, the distances between the five landmarks were consistent across animals and varied <100 μm from mouse to mouse (Figure 1G; n = 19). The consistent depth localization of spike power and CSD sinks and sources during non-REM sleep across animals allowed us to project all subsequent physiological data onto normalized depth coordinates and construct average maps across all animals (Figures 1I and 1J; STAR Methods).

**Figure 2:**
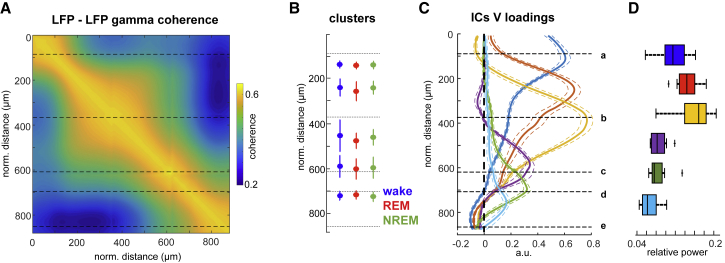
Cortical Layer Identification by LFP Criteria (A) Averaged pairwise gamma (30–100 Hz) LFP coherence during wake for all channels along V1 depth (n = 19 mice). (B) Gradient descent clustering of the cross-coherence matrix separated six putative layers (clusters). Layer boundaries calculated from the gradient decent clusters in all mice and in different brain states (mean ± SEM) are shown. (C) ICA decomposition of gamma band LFPs into six main independent components (ICs). The voltage (V) loadings of ICs are shown as a function of depth (mean ± SEM). Note that the boundaries of ICs correspond approximately to the boundaries predicted from the gamma coherence matrix. Depth landmarks (a–e) are the same as in Figure 1 and apply to (A)–(C). (D) Relative gamma band power for each IC.

To test whether layers could be further disambiguated by the depth localization of LFPs, we examined coherence in the gamma band (30–100 Hz) across all recording sites (Figures 2A and S2). Each site served as a reference, and pairwise coherence with each of the remaining 63 sites was determined iteratively. Using a gradient-descent algorithm (Berényi et al., 2014; STAR Methods), the recording sites were clustered on the basis of their resulting cross-coherence matrix, resulting in six clusters whose five boundaries were consistent across animals when projected into normalized depth coordinates (Figure 2A; p > 0.05, signed rank test for cluster boundaries across animals). The depth distribution of the six gamma coherence clusters corresponded roughly to the six cortical layers in each brain state (Figures 2B and S2; p > 0.05, ANOVA for comparison across different states), suggesting that gamma oscillations in V1 have a highly specific laminar structure. To isolate layer-specific gamma oscillations by another method, we also used independent component analysis (ICA) decomposition of the band-passed (30–100 Hz) LFPs (Fernández-Ruiz et al., 2017; Figure S2). ICA yielded six main independent components (ICs) that were highly consistent across animals (p < 0.001, Spearman’s correlation for ICs spatial loadings), and their depth distribution was related to the anatomically defined layers (Figure 2C). This method has the additional advantage of removing volume-conducted currents (Fernández-Ruiz et al., 2012) that accounted for 0.34 ± 0.11 of V1 gamma LFP variance. The superficial gamma ICs had a significantly larger power compared with the deeper ones (top three versus bottom three; Figures 2D and S4; 0.14 ± 0.04 versus 0.08 ± 0.03 relative power for ICs 1–3 versus 4–6; p = 1.1 × 10^−7^, rank sum test).

### Brain State-Dependent Features of LFPs

**Figure 3:**
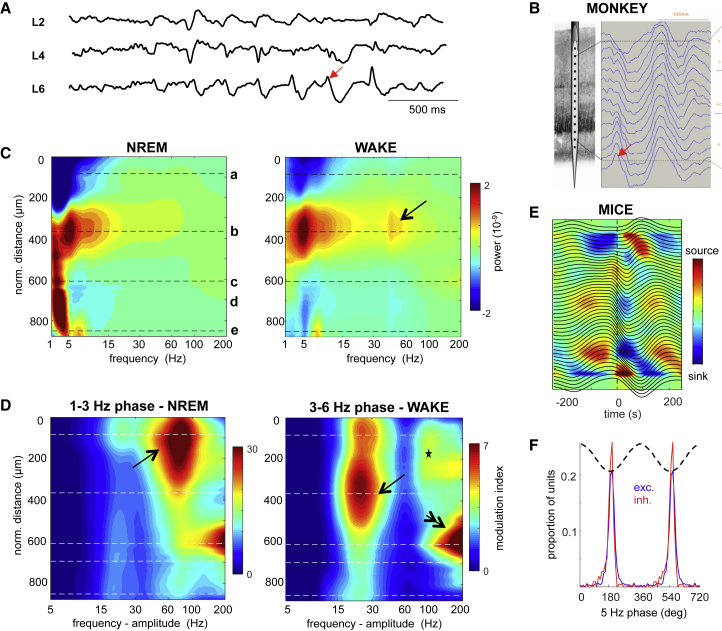
Layer-Specific LFP Patterns in V1 (A) Laminar LFP traces of 3–6 Hz rhythm in the waking, immobile mouse, from layers 2, 4, and 6. (B) For comparison, laminar LFP traces of alpha rhythm in the monkey (reproduced from Bollimunta et al., 2008). Note similar spike and wave waveforms in both cases, as denoted by red arrow. (C) Depth distribution of mean-subtracted LFP power spectra during non-REM sleep and waking. Arrow, slow gamma band in layer 4. (D) On the left, phase-amplitude cross-frequency coupling between the slow oscillation band (1–3 Hz) recorded in layer 6 during non-REM sleep and LFP amplitude across all channels. Note phase coupling to high gamma in superficial layers (arrow). On the right, cross-frequency coupling with the 3–6 Hz LFP phase recorded in layer 4. Note phase coupling of 15–30 Hz (arrow), high gamma (asterisk) in superficial layers, and unit firing-induced high-frequency power in layer 5 (double arrows). (E) CSD and averaged LFP depth profile of the 3–5 Hz rhythm. (F) Spike phase preference distribution of putative principal neurons and interneurons from all layers. Only neurons with significant phase coupling to the 3–6 Hz oscillation were included (p < 0.05, Rayleigh test).

Spectral analysis of the LFP traces revealed depth- and brain state-dependent variation of characteristic frequencies (Figures 3 and S3). Slow oscillation power (0.5–3 Hz) was dominant in non-REM sleep (see Supplementary Information; brain state comparison: p = 4.0 × 10^−4^, Kruskal-Wallis test; n = 19 mice), especially in deeper layers (layer comparison: p = 4.1 × 10^−5^, Kruskal-Wallis test; Figures 3C and S3). Slow oscillation was phase-coupled to gamma frequency power in superficial layers (69 ± 12 Hz; modulation index 2.7 ± 0.5 × 10^−3^) and to the high-gamma (>100 Hz) band in deep layers (Figure 3D; modulation index 2.3 ± 0.3 × 10^−3^). Slow gamma oscillations (30–60 Hz) dominated in superficial layers, especially in the waking state followed by REM and non-REM sleep (Figures 3 and S3; p = 0.028).

Although LFP phase coherence rapidly decreased across layers, especially at higher frequencies (Figure S2A), phase-amplitude cross-frequency coupling revealed reliable interlaminar interactions. In the waking animal, the dominant 3–6 Hz band was phase-coupled to a 15–25 Hz band signal in multiple layers (22 ± 5 Hz; modulation index 4.8 ± 1.2 × 10^−4^), possibly corresponding to the spiky component of the pattern, to high gamma frequency (60–100 Hz) in the superficial layers (90 ± 13 Hz, modulation index 2.7 ± 0.7 × 10^−4^), and to the epsilon band (>100 Hz) in layer 5 (modulation index 6.4 ± 1.8 × 10^−4^), likely reflecting unit firing (Figure 3D).

The most prominent LFP pattern in the waking mouse was a 3–6 Hz oscillation with largest power in layer 4 (Figure 3C, level b; p = 2.0 × 10^−6^, Kruskal-Wallis test; n = 19 mice). This strong power increase was due mainly to frequent epochs of a 3–6 Hz rhythm during waking immobility, in which the waveforms occasionally split into short “spike” and longer “wave” components with a depth profile distribution similar to the depth distribution of alpha waves (8–12 Hz) in monkey V1 (Bollimunta et al., 2008; Figures 3A and 3B). The 3–6 Hz band was cross-frequency phase-coupled to a 15–25 Hz band signal in multiple layers (22 ± 5 Hz, modulation index 4.8 ± 1.2 × 10^−4^), possibly corresponding to the spiky component of the pattern, to high gamma frequency (60–100 Hz) in the superficial layers (90 ± 13 Hz, modulation index 2.7 ± 0.7 × 10^−4^), and to the epsilon band (>100 Hz) in layer 5 (modulation index 6.4 ± 1.8 × 10^−4^), likely reflecting unit firing (Figure 3D).

CSD analysis of the 3–6 Hz rhythm showed a depth distribution of sinks and sources similar to those of the slow oscillation (compare Figure 3E with Figure 1I). The duration of the non-spiking periods of this waking rhythm (50–150 ms) was also similar to the DOWN state of slow oscillation. However, whereas the spiking-associated phase of the waking 3–6 Hz rhythm was short, the duration of the UP state of slow oscillation varied extensively and showed a lognormal distribution (Watson et al., 2016). Both principal cells and interneurons were strongly phase-locked to the trough of the layer 4-recorded 3–6 Hz rhythm (Figure 3F).

Volume-conducted theta power (6–9 Hz) from the hippocampus allowed us to distinguish between waking immobility and ambulation (STAR Methods). As soon as the animal started moving, the 3–6 Hz rhythm vanished, accompanied by increased slow gamma power. The antagonism between movement and the 3–6 Hz oscillation was quantified by the negative correlation between its power and the power of theta oscillation (p < 0.01, signed rank test; Figure S2C). Visual stimulation also blocked an ongoing 3–6 Hz rhythm. However, the offset of visual stimulus often induced a short rebound train 3–6 Hz oscillation (Einstein et al., 2017; Figure S2I).

### Assignment of Neurons to Physiologically Defined Layers

Because “layer” designation is traditionally based on the depth distribution of cell bodies, whereas our depth classification of the LFP patterns (Figure 2) reflects largely a combination of afferent-dendritic excitation and inhibition, we sought to establish a disciplined way to relate LFP-, CSD-, and ICA-based depth estimation to the recorded neurons in different anatomically defined layers.

**Figure 4:**
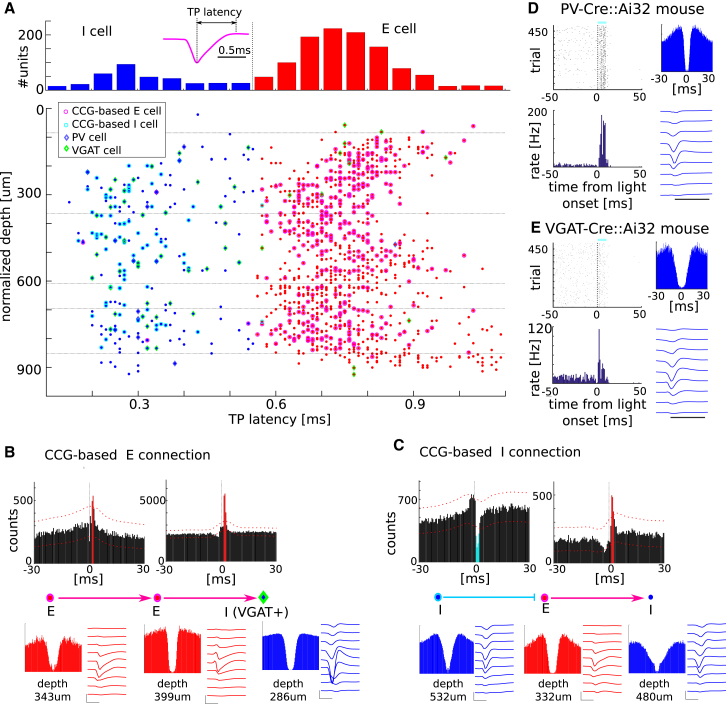
Neuronal Classification on the Basis of Physiological Features in V1 (A) Units (n = 1,472 from 19 mice) were first classified on the basis of trough-to-peak (TP) latency and shown as a function of normalized recorded depth. Each dot corresponds to one unit. Units with trough-to-peak latency < 0.55 ms were tentatively classified as narrow-waveform putative interneurons (I cells; blue). Wide-waveform units were grouped into putative excitatory cells (E cells; red) and inhibitory interneurons (I cells; blue), on the basis of the bimodality of the marginal distribution of TP latencies (top histogram). (B) Example cross-correlograms between a monosynaptically connected E-E-I triplet. Red dotted line, level of significance. The excitatory nature of the postsynaptic neuron is demonstrated by its excitatory action on an identified interneuron. Autocorrelograms and waveforms of each neurons are shown in the bottom raw. (C) I-E-I triplet. Inhibition is inferred from the short (<4 ms) latency suppression of spiking of the postsynaptic cell. Neurons identified as putative E and I cells by short-latency spike cross-correlogram (CCG) are marked by magenta (CCG-based E cells) and light blue circles (CCG-based I cells), respectively, in (A). (D) Example PV-expressing interneuron from a PV-Cre::AI32 mouse in response to blue light activation. (E) Example GABA-expressing inhibitory interneuron from a VGAT-Cre::AI32 mouse in response to blue light activation. PV and VGAT-expressing neurons are marked by blue and green diamonds, respectively, in (A).

Unit clustering was performed as described previously (Fujisawa et al., 2008) using one session from each of the 19 mice, yielding a total of 1,472 units. The physiological identity of clustered units was determined using a multi-step approach (Figure 4). First, the unfiltered waveform was quantified by the trough-to-peak latency of the extracellular spike (Fujisawa et al., 2008, Senzai and Buzsáki, 2017, Niell and Stryker, 2008). This initial classification yielded a bimodal distribution of units with narrow-waveform spikes (putative interneurons, n = 251 I cells) and wide-waveform spikes (putative principal cells, n = 1,075 E cells; Figure 4A; for unclassified units, see Figure S4). We next took advantage of the simultaneously recorded units to physiologically identify them as E cells or I cells by their short-latency temporal interactions with other neurons (Fujisawa et al., 2008). Putative monosynaptic connections are associated with precisely timed spike transmission at short-latency (<4 ms) offsets between two recorded neurons, as detected by narrow significant peaks (excitatory) or troughs (inhibitory) in the cross-correlogram (CCG; Figures 4B and 4C; Alonso and Martinez, 1998, Barthó et al., 2004). Using these criteria, 347 and 100 units were classified as CCG-based E and I cells, respectively (Figure 4A). These CCG-based classification results overlapped well with the waveform-based classification results (97.4% of CCG-based E cells were classified as waveform-based E cells, and 98.0% of CCG-based I cells were classified as waveform-based I cells), supporting the adequate separation on the basis of trough-to-peak latency feature.

We also validated the physiological identification of inhibitory neurons using an optogenetic strategy. A subset of neurons in PV-Cre::Ai32 (n = 5) and VGAT-Cre::Ai32 (n = 9) mice were identified optogenetically as inhibitory neurons (Figures 4D and 4E; blue and green diamonds, respectively, in Figure 4A). Fourteen of 15 optogenetically identified PV cells (n = 11) and 40 of 52 VGAT cells were part of the inhibitory group defined by the spike waveform criterion. These results further support the adequate separation of I cells on the basis of waveform only and also show that a minor fraction of inhibitory neurons (n = 12 of 52) had wider waveforms and were initially falsely combined with the E cell group.

**Figure 5:**
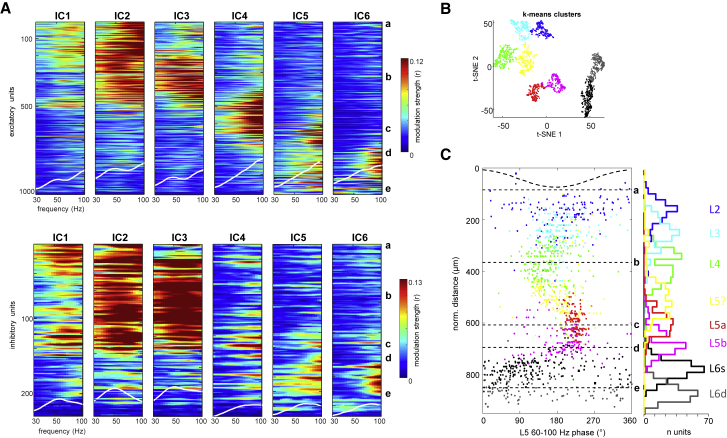
Identification of Cell Body Layers by Spike-LFP Criteria (A) Spike-gamma IC coherence for each principal cell (top) and interneuron (bottom) during waking. For each vertical plot, the reference signal was one of the six ICs. Only units with significant modulation (p < 0.05, Rayleigh test) with gamma band are displayed. Units were sorted according to their depth location. White lines indicate group averages. (B) Spike-gamma IC coupling profile of all V1 units (n = 1,326) in t-distributed stochastic neighbor embedding (t-SNE) space. Eight clusters (color coded) were obtained with K-means. (C) K-means-clustered units are displayed as a function of normalized depth and high-gamma (60–100 Hz) phase preference (reference in layer 5). Circles, principal cells; squares, interneurons. Landmarks a–e are indicated by dashed lines. Right: summed cell density of the eight groups, marked by anatomical cell body labels and putative anatomical layer correspondences. L6A and L6B, superficial and deep sublayers of layer 6. The yellow units do not have traditional cell body layer name (labeled “L5?”).

To link neurons to physiological layers, we examined the relationship between the six gamma ICs and spiking of all neurons. Similar to the LFP-only analysis, spike-IC phase coupling (STAR Methods) also showed clear depth stratification, indicating that units were preferentially modulated by local gamma oscillations (Figures 5A and S5). Around and above depth level b (i.e., layer 3/4 border; first three components), unit-IC coupling of both putative E and I neurons had a peak in the slow gamma range. In deep layers, unit-IC coupling shifted toward the fast gamma band. This fast gamma (60–100 Hz) phase preference also segregated E and I neurons along the cortical depth (Figure S5). We therefore used the spike-gamma phase coupling profile of all units against the six ICs to perform K-means clustering (STAR Methods). This procedure yielded eight clusters (Figure 5B). By projecting back the K-means-clustered units to cortical depth, we found that the clusters largely corresponded to the anatomically defined cell body layers (Figure 5C). The top two clusters may correspond to neurons in layers 2 and 3 (blue and cyan), and the third cluster surrounded landmark b (green) and corresponded to the layer 3/4 border sink (Figure 1I). Three clusters surrounded landmark c (yellow, red, and pink), the presumed center of layer 5, with the lowest of the three clusters surrounding landmark d, the deep sink, likely corresponding to layer 5/6 (Reyes-Puerta et al., 2015). The bottom two clusters (black and gray) were designated as layer 6 neurons. The remaining unit analyses were performed on these clustered groups of neurons.

### Layer-Specific and Brain State-Dependent Features of Unit Firing

**Figure 6:**
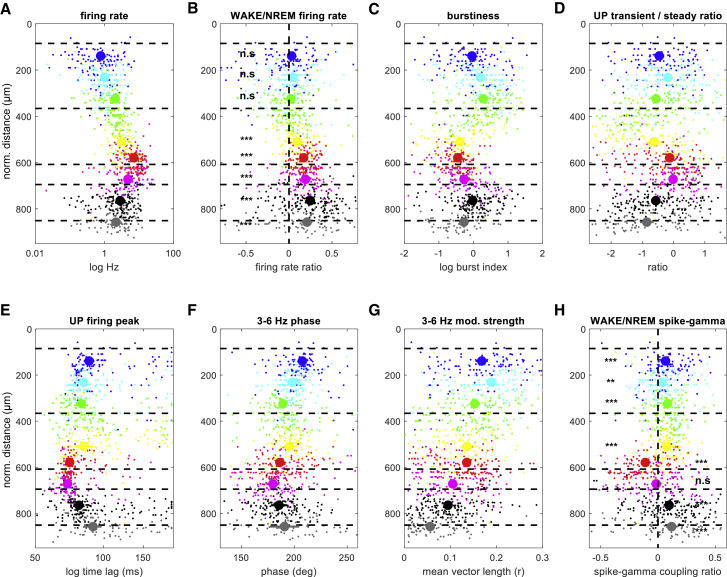
Physiological Properties of Neurons in Different Layers (A) Log firing rates in no-REM sleep. Colors correspond to the eight clusters of Figure 5C. Large circles, group means. Landmarks a–e are indicated by dashed lines. (B) Firing rate ratios between waking and non-REM sleep. Significant differences between states are indicated for each group. (C) Log burst index of units as a function of depth. (D) Transient-steady ratio of firing rate during UP state of non-REM sleep. (E) Latency of spikes after DOWN-UP transitions during non-REM sleep. (F) Spike-phase preference to the 3–6 Hz LFP rhythm recorded in layer 4. (G) Mean resultant length (modulation strength) of units during 3–6 Hz rhythm. (H) Brain state-dependent changes of spike-gamma LFP coupling between wake and non-REM. ^∗∗^p < 0.01 and ^∗∗∗^p < 0.001 (rank-sum test).

The various physiological features of the recorded principal neurons as a function of depth are summarized in Figure 6 (see Figure S6 for interneurons). Long-term mean firing rates of V1 principal cells showed a strongly skewed distribution in each layer. The fastest firing group corresponded to those in layers 5A and B (Figure 6, red and magenta groups), while the slowest ones were the superficial layers (Figure 6A; p = 3.7 × 10^−83^, Kruskal-Wallis test). Deep-layer neurons were significantly more active during waking compared with non-REM sleep (Figure 6B; see Figure S6 for other state comparisons) (Sakata and Harris, 2012, Petersen and Crochet, 2013).

A characteristic physiological feature of cortical pyramidal neurons is their bursting property (Nowak et al., 2003). Bursting was determined by calculating the number of spikes in the 3–10 ms bins of the spike autocorrelograms divided by the number of spikes in the 200–300 ms bin. In general, superficial layer neurons exhibited more bursting behavior, followed by layer 6 and layer 4 neurons, while layer 5 neurons had the lowest burst indices (Figure 6C; p = 5.2 × 10^−37^, Kruskal-Wallis test). Another measure of spike dynamic is the relationship of the neuron’s initial phasic response versus sustained firing to a given input. We calculated this transient/steady firing rate index by dividing the peak firing rates at 0–200 ms by the mean firing rates at 100–200 ms after the UP state onset of slow oscillation (Figures 6D and S2C). By this measure, deep layer 5 neurons showed the strongest transient responses (layer difference, p = 5.6 × 10^−10^). During the DOWN-UP state transition, layer 5B neurons fired first, followed by superficial and layer 6 neurons (Sakata and Harris, 2009, Beltramo et al., 2013; Figure 6E; p = 1.3 × 10^−38^). The distribution of spike firing latency during the 3–6 Hz rhythm of waking displayed characteristic laminar differences (Figure 6F; p = 2.6 × 10^−10^), similar to spike latencies during slow oscillations. The mean vector length of phase locking to the 3–6 Hz rhythm was strongest in the superficial layers and weakest in layer 6 (Figure 6G; p = 7.6 × 10^−50^). Spike-gamma (30–100 Hz) coupling was stronger in the waking animal compared with non-REM in all layers but layer 5A (Figure 6H).

The strongest spike-LFP coupling was observed in the 3–6 Hz band (Figures 3F and S7), followed by the slow gamma (30–60 Hz) band, each with characteristic cortical depth and brain state-dependent profiles (Figure S7).

### Within-Layer and Inter-layer Interactions among Neurons

**Figure 7:**
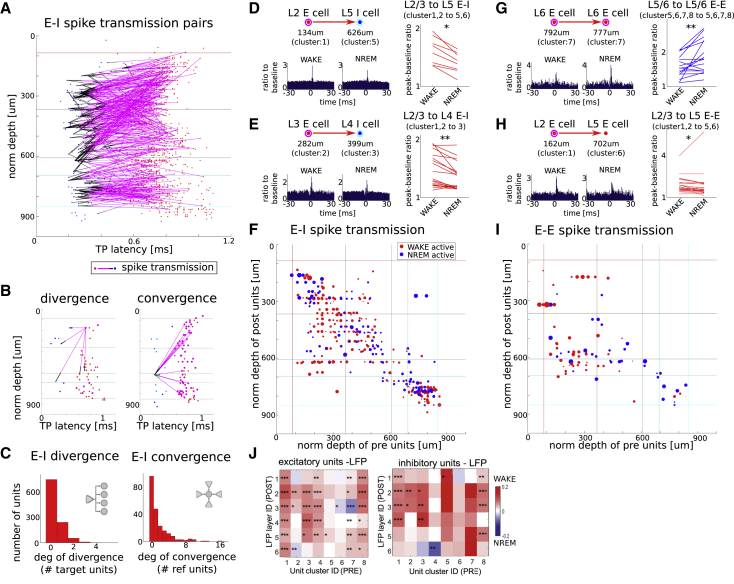
Spike Transmission Probability Changes across Brain States (A) E-I monosynaptic connections. Putative inhibitory (blue) and excitatory (red) unit pairs are shown as a function of recording depth and trough-to-peak (TP) latency of units. Magenta lines with black ends indicate putative monosynaptic pairs from E to I neurons. (B) Examples of E to I divergence and convergence from two different mice. (C) Distributions of E-I divergence and convergence. (D and E) E-I spike transmission probability change across waking and non-REM sleep states in an example pair (left) and all pairs (right) for L2/3 to L5 (D) and L2/3 to L4 (E). (F) Comparison for all E-I pairs. The sizes of the dots indicate the magnitude of difference between states. Red, stronger transmission during wake; blue, stronger transmission during non-REM sleep. (G and H) E-E spike transmission probability change across waking and non-REM sleep states for L5/6 to L5/6 (G) and L2/3 to L5 (H). (D–H) ^∗^p < 0.05, ^∗∗^p < 0.01 signed-rank test. (I) Comparison for all E-E pairs. (J) Changes between waking and non-REM sleep in spike-gamma LFP coupling ratio for E and I neurons. ^∗^p < 0.05, ^∗∗^p < 0.01, and ^∗∗∗^p < 0.001 (rank-sum test). Neuron groups 1–8 are as in Figure 5C.

Of the 102,332 potential neuron pairs in all mice and sessions, we identified putative excitatory (E, n = 525) and inhibitory (I, n = 323) monosynaptic connections by the spike transmission probability method (Figures 7 and S7). This is a strongly under-sampled estimate, because the spike transmission probability-based method requires simultaneous recordings of spikes of both pre- and postsynaptic neurons, and large numbers of spikes are needed to detect weak synapses (Galarreta and Hestrin, 2001, Schwindel et al., 2014, English et al., 2017). Although many identified excitatory connections were detected within the same layer (layers in these calculations refer to IC-based clusters) for both E-I (Figures 7A and 7B; n = 173 of 385 connections) and E-E (n = 19 of 90) pairs as well as inhibitory I-E pairs (Figure S8; n = 98 of 323 connections), there were also many cross-layer excitatory and inhibitory pairs (I-I pairs were too few to draw meaningful conclusions). A considerable fraction of layer 2/3 principal cells had monosynaptic connections with layer 4 and 5 interneurons (54.7%) and principal cells (22.7%) (Figures 7E and 7G). Of the 172 putative excitatory connections of L2/3 pyramidal cells, 10 (5.8%) and 39 (22.7%) targeted L2/3 and L4/5/6 excitatory neurons, respectively, in addition to contacting 29 (16.9%) and 94 (54.7%) L2/3 and L4/5 interneurons, respectively. Most individual presynaptic principal neurons had only a single partner target in the recorded population but a small fraction targeted multiple partners (Figures 7B and 7C). A typical interneuron typically had one presynaptic partner, but a small fraction many, up to 16 (Figure 7C). Almost half of the E-I connections (168 of 392 [42.9%]) received reciprocal I-E connections when all layers were considered. Reciprocal E-I-E connections were almost twice more frequent in layer 5 (60%–70%) than in other layers (Figure S7C).

To quantify the brain state-dependent changes in the spike transmission probabilities, we calculated the peak-to-baseline ratio of CCGs (Fujisawa et al., 2008, Senzai and Buzsáki, 2017) between E-I cells (Figures 7D and 7E) and between E-E cells (Figures 7F and 7G) as well as between I-E pairs (Figure S8) in different brain states. We observed that spike transmission strength between layer 2/3 E cells and deep layer I and E cells became weaker during non-REM sleep compared with waking (L2/3 E cells to L5 I cells, p = 0.0012; L2/3 E cells to L4 I cells, p = 0.0010; and L2/3 E cells to L5 E cells, p = 0.015; signed rank test; Figures 7D–7F, 7H, and 7I). In contrast, putative E-E spike transmission in the deep layers (layer 5/6 to layer 5/6 principal cells) significantly increased during non-REM sleep compared with waking (p = 0.0061, signed rank test; Figures 7G and 7I). These results indicate that communication from superficial to deep layers increases during waking, while excitatory interactions become stronger within deep layers during non-REM sleep. These findings were also reflected by unit-LFP coupling when all eight unit clusters were compared with the six gamma ICs (Figure 7I). When all I-E pairs were considered, spike suppression probability during waking was significantly stronger compared with non-REM (I-E dip wake, 24.1%; non-REM sleep, 22.8% relative to the CCG baseline; p = 0.006, signed rank test; Figure S8F).

### DOWN State-Active Neurons

**Figure 8:**
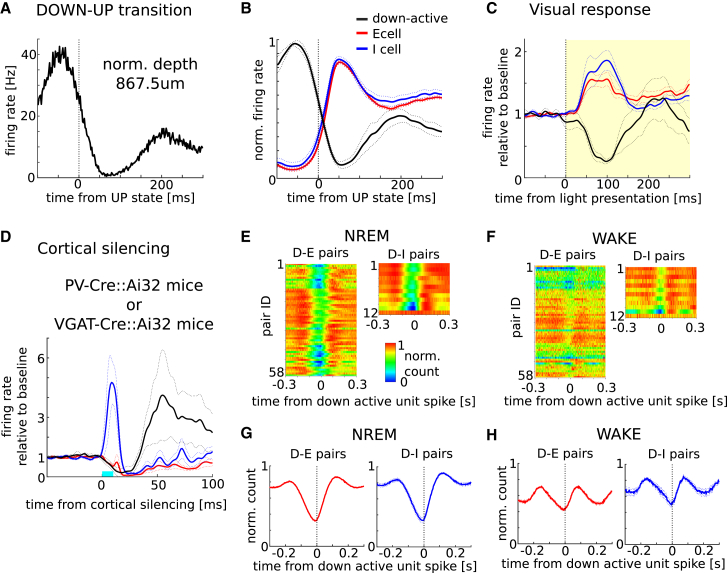
DOWN State-Active Neurons (A) Histogram of an example neuron. Time zero corresponds to the DOWN-UP state transition of slow oscillation. (B) Normalized group firing rate histograms for all principal cells (red, E), interneurons (blue, I), and five DOWN state-active neurons (black; n = 4 mice; mean ± SE). (C) Light-evoked activity of principal cells, interneurons, and a DOWN state-active neuron from an example session (n = 500 repetitions; mean ± SEM). (D) Suppression of spiking activity by optogenetic activation of interneurons (blue line) for all principal cells, interneurons, and five DOWN state-active neurons (mean ± SEM). (E and F) Spike cross-correlations between a DOWN state-active neuron and all other principal cells (D-E pairs) and interneurons (D-I pairs) during non-REM sleep (E) and waking (F) from a single mouse. Color code, normalized counts. (G and H) Cross-correlations between spikes of five DOWN state-active neurons and spikes of all other principal cells and interneurons during non-REM sleep (G) and waking (H); n = 4 mice.

We found five neurons in four mice that were specifically and dominantly active during the DOWN state of non-REM slow oscillation (Figures 8A and 8B). All DOWN state-active neurons were found in deep layers 6. Four neurons had bursty autocorrelograms, while the fifth one was regular spiking. These five neurons were not only active during the DOWN state but mainly silent during the UP state (35.2 ± 23.1 Hz during DOWN, 4.3 ± 3.1 Hz during UP; p < 0.016, rank sum test). During light flash presentation, their spiking activity was further reduced at the time when the remaining neurons robustly responded (Figure 8C). During optogenetic activation of PV neurons or VGAT neurons, the DOWN state-active neurons were also suppressed, indicating that at least some classes of interneurons innervate them. However, ∼30 ms after the optogenetic stimulation, they rebounded to high level of activity, while the remaining population of principal neurons was still suppressed (Figure 8D). Cross-correlation between the DOWN state-active cells and other principal cells showed an inverse correlation not only during non-REM but also in the waking animal (Figures 8E–8H). In summary, the rare DOWN state-active neurons showed a robust inverse correlation with the activity of all other neurons during both sleep and waking.

## Discussion

Depth profiles of unit power and sink-source distributions of slow oscillations of non-REM sleep provided consistent physiological landmarks in V1 across animals. We report the following findings: (1) Coherence and ICA of gamma oscillations (30–100 Hz) and spike-gamma LFP coupling identified six physiological layers and distinguished further sublayers. (2) Firing rates, burstiness, and other physiological features of neurons displayed distinct layer and brain state dependence. (3) Monosynaptic connections, quantified by a spike transmission probability method, revealed highly structured distributions within and across layers. Connection strengths were skewed, with a minority of highly connected hubs. Spike transmission between E-E pairs and E-I pairs from layer 2/3 to layer 5 was stronger during waking compared with non-REM sleep but stronger among deep-layer excitatory neuron pairs during non-REM sleep. (4) The most prominent LFP pattern during waking was a 3–6 Hz rhythm with characteristic phase preferences of spikes across layers. (5) Spiking of a small subset of neurons in deep layers was anticorrelated with all other neurons, and these neurons were most active in the DOWN state of slow oscillations. Our findings link mesoscopic LFPs and single-neuron interactions with multilayered anatomical organization in V1.

### Physiological Identification of Functional Layers in the Neocortex

The term “layer” has been used differently in different structures. For example, in the hippocampus, apical and basal dendritic layers are distinguished from somatic layers of the same neurons. In contrast, “layer” in the neocortex traditionally refers to histologically distinct somatic layers. However, a given somatic layer (e.g., layer 2) is also the apical dendritic layer of other neurons (e.g., layer 3 and layer 5 neurons). Afferents from different upstream regions can preferentially target neurons of a given layer or multiple layers. For example, although the highest density of thalamocortical afferents is present in layer 4, collaterals of these axons also innervate both superficial and deep layers, contacting several types of neurons (Harris and Shepherd, 2015). Nevertheless, afferents from different sources typically segregate on different segments of dendrites (Bock et al., 2011). Similarly, inhibitory interneurons of the same class converge on the same neuronal domains, whereas different types converge on distinct somadendritic domains of their targets. In addition, biophysical experiments demonstrate that different segments of pyramidal neurons are electrically isolated, and each functional class of inputs is initially processed in relative independence of the other (Larkum et al., 1999). Our experiments identified these physiologically distinct layers.

Previous work in the hippocampus has shown that coherence in the gamma frequency range, measured across electrodes in the same layer innervated by the same afferents, is high over long lateral distances, whereas coherence across electrodes placed just 100 μm apart but in different layers is low (Fernández-Ruiz et al., 2017). Similar segregation by coherence has been described between superficial and deep layers in the V1 of the monkey (Maier et al., 2010). We hypothesize that segregation of excitatory and inhibitory inputs on the orderly arranged principal cell populations may be responsible for the observed layer-specific extracellular gamma currents (Fernández-Ruiz et al., 2017).

Both the gradient-descent algorithm performed on gamma oscillation and ICA identified six strata, which can be regarded as functionally distinct layers. Spike-gamma LFP phase-coupling, in turn, allowed us to relate these physiological layers precisely to the depth distribution of neuronal somata. This additional step effectively separated layer 6 neurons into deep and superficial groups and divided layer 5 neurons into three subgroups. By depth criteria, two of these three groups may be classified as layer 5A (cortico-cortical with thin apical dendrites) and 5B (cortical-subcortical with thick apical dendrites) neurons, respectively (Harris and Shepherd, 2015). Our third, most superficial group may represent a transitional form between layer 4 and layer 5 neurons. Our clustering method also identified two groups in layers 2/3, possibly corresponding to layer 2 and layer 3 neurons. Our physiology-based classification of principal neurons will require confirmation by future optogenetic experiments using available genetic markers of layer and sublayer-specific pyramidal neurons (Gouwens et al., 2018). Overall, our findings demonstrate that physiological properties of neurons, especially their relationship to gamma LFP, can be exploited to relate them to classical anatomy-based layer segregation. In turn, the spiking activity of the classified groups can be examined for their contribution to brain state-dependent collective network patterns.

### 3–6 Hz Oscillation

The most prominent LFP pattern in the waking V1 was a 3–6 Hz oscillation. We hypothesize that this rhythm is an evolutionary precursor of the primate alpha activity in the visual cortex. First, the 3–6 Hz oscillation is the dominant rhythm in the waking mouse as is the case for the 8–12 Hz alpha oscillation in the primate. Its power is highest in layer 4, and its current sink-source depth distribution is similar to the alpha waves in the monkey (Dougherty et al., 2017, Bollimunta et al., 2008, Bollimunta et al., 2011). Another similarity to alpha oscillations is the strong phase-power and power-power coupling between 3–6 Hz and gamma oscillations (Osipova et al., 2008). Second, the behavioral correlates of the 3–6 Hz oscillations are similar to alpha. Both are most prominent during quiet waking, occur in several seconds-long bouts, and are reduced or eliminated by movement and arousal and reduced in non-REM sleep (Klimesch et al., 2007, Arroyo et al., 2018; present observations). Yet their probability can be increased by the offset of visual stimulation in the mouse (Einstein et al., 2017; present findings) and rat (Pickenhain and Klingberg, 1967) and by milk reward in the cat (Buchwald et al., 1964). When visual cues are presented during the 3–6 Hz oscillations, the responses of V1 pyramidal neurons are reduced (Einstein et al., 2017, Arroyo et al., 2018), similar to attenuated visual evoked responses in humans (Klimesch et al., 2007). Intracellular recording in layer 2/3 pyramidal cells, parvalbumin and somatostatin-expressing neurons in V1 of mice revealed large amplitude, rhythmic hyperpolarizations at 3–6 Hz after the offset of visual stimulation, and spiking between the hyperpolarization (Einstein et al., 2017), as observed for interneurons, including parvalbumin-expressing cells in our study. Similar rhythmic hyperpolarizations were observed in layer 2/3 pyramidal neurons at 2 Hz under anesthesia and blocked by visual stimulation (Sun and Dan, 2009). Third, we observed the strongest spike-LFP coupling in all cortical layers in the 3–6 Hz band. The earliest and strongest firing was present in layers 4 and 5B, coinciding with the LFP sink in layer 4 followed by neurons in the superficial layers, similar to neuronal patterns during alpha oscillations in different cortical layers in the monkey (Dougherty et al., 2017, Bollimunta et al., 2008, Bollimunta et al., 2011).

We found similar sink-source distributions during the 3–6 Hz rhythms and slow oscillations of non-REM sleep. It is generally believed that the neuronal substrate of several slow-frequency oscillations in the neocortex is the thalamocortical resonant network and that the distinct patterns, such as slow oscillations, alpha rhythm, mu rhythm, and sleep spindles, and their timing circuits are set largely by the combination of subcortical neuromodulatory inputs affecting both thalamic and neocortical targets (Steriade et al., 1993, Lörincz et al., 2009, McCormick and Bal, 1997, Crunelli and Hughes, 2010). The increased tone of cholinergic and monoaminergic afferents during movement, arousal, and visual stimulation are known to suppress thalamocortical oscillations (McCormick and Bal, 1997), including alpha oscillations in humans (Osipova et al., 2008), 6–9 Hz thalamocortical oscillations in rats (Buzsáki et al., 1991), and the 3–6 Hz rhythm in mice (Pinto et al., 2013). In sum, although the frequency of the primate alpha is more than twice higher than the 3–6 Hz rhythm in the mouse, many of their physiological features are remarkably similar. In support of the evolutionary conservation hypothesis, the frequency of the V1 rhythm increases with brain size from mouse (4 Hz; Einstein et al., 2017) to cat (6–12 Hz; Buchwald et al., 1964, Lörincz et al., 2009) to human (8–12 Hz; Klimesch et al., 2007).

### State Dependence of Firing Patterns and Interlaminar Interactions

Both firing rates and bursting properties of principal cells varied across different layers. In agreement with previous observations in different cortical areas and species, layer 2/3 pyramidal neurons had the lowest median firing rates, followed by layer 4 and layer 6 neurons, whereas the highest firing rate cells resided in layer 5 (Sakata and Harris, 2012, Petersen and Crochet, 2013). The lower firing rates of layer 2/3 excitatory neurons may, at least in part, result from their resting membrane potentials being ∼10 mV hyperpolarized relative to superficial layer pyramidal neurons, according to *in vitro* measurements (Petersen and Crochet, 2013). Layer 2/3 pyramidal neurons may therefore require substantially more excitatory synaptic input to drive them to action potential threshold compared with L5 pyramidal neurons. Deep-layer neurons increased their excitability during waking (Sakata and Harris, 2012), as quantified by their firing rates, whereas no such state dependence was present in superficial neurons.

Layer 5 neurons are often segregated into intrinsically bursting and regular firing neurons on the basis of in response to intracellular current injection (Silva et al., 1991). In our experiments, we found that layer 5 neurons, in general, had the lowest propensity to induce spike clusters at short interspike intervals. On the other hand, layer 5 neurons had the highest index of transient/steady ratio during the UP state of slow oscillation, reminiscent of the neuron’s response to sustained intracellular depolarization (Nowak et al., 2003).

We identified monosynaptic connections between pairs of neurons both within and across layers using short-term spike transmission probability (Fujisawa et al., 2008, Schwindel et al., 2014, Alonso and Martinez, 1998). The magnitude of both spike transmission probability and single neuron-induced excitatory postsynaptic potentials (EPSPs) (in E-I and E-E connections) and IPSPs (I-E connections) showed a lognormal distribution (Song et al., 2005), implying that a very small number of strong connections are mixed with a large number of weak connections. Detection of monosynaptic connections by the spike transmission probability method depends on the combination of the strength of the synapse and the number of spikes available in a given recording session (Schwindel et al., 2014), therefore it is biased toward neuronal pairs with stronger connections, such as E-I connections and I-E pairs (English et al., 2017, Branco and Staras, 2009). This relationship may explain why only a small fraction of all possible E-E connections (Schwindel et al., 2014, Cossell et al., 2015) was detected and why more E-I than E-E pairs were identified in our study. Yet our under-sampled population can still be regarded as representative for the relative distribution of the connectivity features.

Surprisingly, most E-I and I-E connections were across layers, including deep-to-superficial and superficial-to-deep links. Layer 2/3 neurons innervated >3 times as many layer 4/5 principal cells and interneurons as their peers in the home layer. Connection strength tended to cluster around a few hub neurons (Fujisawa et al., 2008, English et al., 2017, Bock et al., 2011, Song et al., 2005). We also found that reciprocal connections between E and I neurons (Song et al., 2005) varied across layers and were almost twice as frequent in layer 5 compared with other layers. These findings indicate that cortical connectivity is highly structured, and interlaminar interactions are fundamental to multi-layer cortical operations (Song et al., 2005). The use of higher density, multishank probes in future experiments will undoubtedly increase the yield of synaptically connected neurons and provide more reliable quantification than we found in the present experiments. This expected progress is facilitated by the fact that the number of neuron pairs increases as the square of the number of units recorded.

Spike transmission probability between superficial and deep layers was stronger in the waking animal for both E-I and E-E pairs, compared with non-REM sleep. This finding, combined with the suggestion that thalamocortical synapses are not affected by brain state (Stoelzel et al., 2009), supports the hypothesis of top-down control of visual processing (Gilbert and Li, 2013, Bastos et al., 2015). In contrast, spike transmission strength increased between E-E pairs and I-E pairs during non-REM sleep, preserving the E-I balance (Atallah and Scanziani, 2009) even during the UP state of slow oscillation, associated with strong recurrent excitation of deep layer pyramidal neurons (Sanchez-Vives and McCormick, 2000). The present study compared only distinct brain states, such as waking and sleep. However, the translaminar recordings combined with quantified spike transmission probability measures can be also used to monitor within and interlaminar interactions in visual or other cortical areas for studying cortical processing in perception, learning, and motor planning.

### DOWN State-Active Neurons

A small subset of layer 6 neurons showed a robust inverse correlation with the activity of other principal cells and interneurons in all brain states, which was particularly striking during non-REM sleep. We named these cells DOWN state-active neurons because they are specifically and selectively active during the DOWN state of slow oscillations (Steriade et al., 1993). The identity of the DOWN-state active neuron is not known at present. On the basis of their location and brain state correlates, they might correspond to a subset of GABAergic interneurons that express neuronal NOS (nNOS). These nNOS-expressing neurons increase their c-Fos expression during non-REM sleep and are distributed mostly in deep cortical layers (Gerashchenko et al., 2008). Optogenetic identification and manipulation of these neurons in future experiments may reveal their exact physiological contribution to slow oscillations and other network functions.

## STAR★Methods

### Key Resources Table

**Table:** 

REAGENT or RESOURCE	SOURCE	IDENTIFIER
**Chemicals, Peptides, and Recombinant Proteins**		

DAPI	Sigma-Aldrich	D9542 SIGMA
NeuroTrace 500/525 Green Fluorescent Nissl Stain	Thermo Fisher Scientific	N21480

**Deposited Data**		

Electrophysiology data	Buzsáki lab	http://buzsakilab.com/wp/datasets/

**Experimental Models: Organisms/Strains**		

Mouse: B6;129S-Gt(ROSA)26Sortm35.1(CAG-aop3/GFP)Hze/J	The Jackson Laboratory	JAX: 012735
B6;129P2-Pvalbtm1(cre)Arbr/J	The Jackson Laboratory	JAX: 008069
B6;129S-Gt(ROSA)26Sortm32(CAG-COP4^∗^H134R/EYFP)Hze/J	The Jackson Laboratory	JAX: 012569
B6.Cg-Tg(Camk2a-cre)T29-1Stl/J	The Jackson Laboratory	JAX: 005359
B6J.129S6(FVB)-Slc32a1tm2(cre)Lowl/MwarJ	The Jackson Laboratory	JAX: 028862

**Software and Algorithms**		

Analysis tools	Buzsáki lab	https://github.com/buzsakilab/buzcode
MATLAB	MathWorks	https://www.mathworks.com/
Kilosort	Pachitariu et al., 2016	https://github.com/cortex-lab/KiloSort
Phy	Kwik Team	https://github.com/kwikteam/phy
ICA algorithms	EEGLAB	https://sccn.ucsd.edu/eeglab/
Wavelet analysis toolbox	Torrence and Compo, 1998	http://atoc.colorado.edu/research/wavelets/

**Other**		

Silicon probe: 1shank (H3)	Cambridge NeuroTech	https://www.cambridgeneurotech.com/silicon-probes
Intan RHD2000	Intan Technologies	http://intantech.com/RHD2000_evaluation_system.html
450nm laser diode	Osram Laser Diodes	PL450B
520nm laser diode	Osram Laser Diodes	PL520B
LD current controller	Thorlabs	LDC202C

### Contact for Reagent and Resource Sharing

Further information and requests for reagents and resource may be directed to, and will be fulfilled by the Lead Contact, Dr. György Buzsáki (gyorgy.buzsaki@nyumc.org).

### Experimental Model and Subject Details

All animal handling procedures were approved by the Institutional Animal Care and Use Committee of New York University Medical Center. We used several lines of transgenic mice Mice (n = 19; n = 3 Ai35; n = 5 PV-Cre::Ai32; n = 9 VGAT-Cre::Ai32; n = 1 CaMKII-Cre::Ai35; n = 1 CaMKII-Cre::Ai32) for optogenetic tagging of the recorded units.

### Method Details

#### Surgery and electrode implantation

A total of 19 male mice (28-35 gr, 3-8 months old) were implanted with recording electrodes under isoflurane anesthesia. These procedures were performed in two steps.

In the first step, a ground electrode (100-μm diameter tungsten wire) was implanted in the contralateral cerebellum and head plate base was placed around the implantation target area (the primary visual cortex, V1). After 13 day of recovery from the first step, mice underwent the silicon probe implantation procedure. A single shank silicon probe (Cambridge NeuroTech H3 64x1 probe) was mounted on a movable microdrive for recording the activity of multiple single-units and local field potentials (LFPs) in V1. The high-density silicon probe had 64 recording sites (100k–1MΩ impedance each site), aligned on the linear edge of the probe (20-μm vertical separation). After the craniotomy above the target implantation site, the probe was implanted at anteroposterior: +1.0mm, mediolateral: +2.5 mm, with a 21° angle from dorso-ventral axis and a 10° angle from the mediolateral axis, so that the probe was perpendicular to the brain surface at the target site. The probe was lowered to 1.0 mm below the brain surface. For optogenetic tagging of specific cell types, an optic fiber was placed right above the skull over the implantation site. The back end of the fiber was coupled to a laser diode (450 nm blue, Osram) (Stark et al., 2012).

After the second operation (< 30 min), mice were allowed to recover overnight in their home cage before the recording session.

#### Extracellular electrophysiological recording

We recorded from the mice while they slept or walked around freely moving in the home cage for of 68 hr. Electrophysiological data were acquired using an Intan RHD2000 system (Intan Technologies LLC) digitized with 20 kHz rate. The wide-band signal was down-sampled to 1.25 kHz and used as the LFP signal.

For optogenetic tagging of specific neuron types, blue laser light pulses were delivered above the V1. The maximum light power at the tip of the optic fiber was 1 to 3 mW (450 nm, Osram Inc). 10 ms light pulses with 40%, 70% and 100% of the maximum power were delivered (n = 500 times at each intensity, 1 Hz). At the beginning or the end of the recording session, light from laser diode (520 nm, Osram) was delivered in the home cage to induce light-evoked responses of V1 neurons (n = 500 times, 400 ms duration pulses, 0.5 Hz).

#### Electrolytic lesions

In four mice, a small current was delivered through two of the recording sites to produce an electrolytic lesion for subsequent histological verification of the recording depths. The mouse was anesthetized with isoflurane and placed in a stereotaxic apparatus. A thin metal bar (anal electrode) lubricated with vaseline was inserted in the anus of the animal and 4 to 10 μA current was applied between the anal electrode and target electrode channel on the silicon probe for 5-10 s per channel. After electrolytic lesioning procedure, mice were returned to their home cage. After two days, the animal was sacrificed.

#### Histological processing

At the end of the recording session or two days after the electrolytic lesion, mice were overdosed with pentobarbital injection (100 mg/kg body weight), perfused with saline and 4% paraformaldehyde before their brains were rapidly removed. After overnight post fixation in 4% paraformaldehyde solution, the brain was washed in PBS three times. Coronal sections (50 μm) were cut on a vibratome (Leica, VT1000S) and brain slices were collected in PBS. To identify the borders between neocortical layers, fluorescent Nissl staining was performed. The procedure consisted of one time 10 min wash with PBS-0.1% Triton solution (PBS-T) for permeabilization, 20 min incubation in NeuroTrace 500/525 Green Fluorescent Nissl Stain diluted by 300-fold, two times of 10 min wash in PBS-T, three times of 5 min wash in PBS, and 2 hr wash in PBS. After the washing procedures, brain sections were mounted in Fluoromount with DAPI (Sigma) and imaged with a confocal laser-scanning microscope (Zeiss, LSM 700).

#### Spike sorting

Spike sorting was performed semi-automatically, using Kilosort (Pachitariu et al., 2016). This was followed by manual adjustment of the waveform clusters using the software Phy. Following parameters were used for the Kilosort clustering.

ops.Nfilt 6 ^∗^ numberChannels
ops.nt0 64
ops.whitening ‘full’
ops.nSkipCov 1
ops.whiteningRange 64
ops.criterionNoiseChannels 0.00001
ops.Nrank 3
ops.nfullpasses 6
ops.maxFR 20000
ops.fshigh 300
ops.ntbuff 64
ops.scaleproc 200
ops.Th [4 10 10]
ops.lam [5 20 20]
ops.nannealpasses 4
ops.momentum 1./[20 800]
ops.shuffle_clusters 1
ops.mergeT 0.1
ops.splitT 0.1
ops.initialize ‘no’
ops.spkTh 31

#### Detection of monosynaptic functional connectiveties

Cross-correlograms of spike trains of neuron pairs can reveal putative synaptic connections between them (Fujisawa et al., 2008, Barthó et al., 2004, Stark and Abeles, 2009). This takes the form in the cross-correlogram of short time-lag (1–4 ms) positive or negative deviations from baseline indicating putative excitatory or inhibitory connections, respectively. Such detection is based on testing the null hypothesis of a homogeneous baseline at short time-scale (Stark and Abeles, 2009). To this end, cross-correlograms binned in 0.5-ms windows were convolved with a 7-ms standard deviation Gaussian window resulting in a predictor of the baseline rate. At each time bin, the 99.9999 percentile of the cumulative Poisson distribution (at the predicted rate) was used as the statistical threshold for significant detection of outliers from baseline. A putative connection was considered significant when at least two consecutive bins in the cross-correlograms within +1.5 to +4 ms passed the statistical threshold. Corrected spike transmission probability was calculated as described in English et al. (2017).

#### Classification of units based on spike waveforms

Units with maximum waveform amplitude with positive sign were classified as ‘positive waveform units’, and those with negative sign as ‘negative waveform units’. Putative fiber potential was identified by the kurtosis of the normalized waveform (maximum amplitude = 1), calculated as the second derivative of negative waveform units. If the kurtosis was larger than ± 5SD of the mean, they were classified as putative fiber units. For the remaining negative waveform units, units with the trough-to-peak latency (TP latency) > 0.55 ms were tentatively classified as putative E cells and those with TP latency ≤ 0.55 ms were classified as putative I cells.

#### Optogenetic tagging of PV cells and VGAT cells

To optogenetically tag PV cells and VGAT cells in V1, PV-Cre::Ai32 mice and VGAT-Cre::Ai32 mice were used, respectively. 10 ms light pulses were delivered every 1 s for 500 times. Light-triggered post-event histogram was binned at 1 ms width. If the peak firing rate in a 1-6 ms window after the light delivery (average of the first and second maximum value) was larger than the ± 8 SD of the mean of the baseline (–100-0 ms before the light delivery), the unit was defined to be optogenetically activated.

#### Data analysis

##### Brain state scoring

Spectrograms for brain state scoring were constructed with a 1 s sliding 10 s window FFT of 1250 Hz LFP data at log-spaced frequencies between 1 and 100 Hz. Three types of signals were used to score state in our recordings: broadband LFP, narrowband theta frequency LFP, and electromyogram (EMG). For broadband LFP signal, principal components analysis (PCA) was applied to the z-transformed (1-100 Hz) spectrogram. The first PC in all cases was based on power in the low (< 20 Hz) frequency range and had oppositely weighted power in the (> 32 Hz) higher frequencies. Theta dominance was taken to be the ratio 5-10 Hz and 2-16 Hz power from the spectrogram. EMG was extracted from the intracranially recorded signals by detecting the zero time-lag correlation coefficients (r) between 300-600 Hz filtered signals (using a Butterworth filter at 300 – 600 Hz with filter shoulders spanning to 275 – 625 Hz) recorded at all sites (Schomburg et al., 2014). The state scoring algorithm was performed by a series of divisions with thresholds set at the trough between the peaks of distributions in these three metrics (Watson et al., 2016) (https://github.com/buzsakilab/buzcode/tree/master/detectors/detectStates/SleepScoreMaster). First, non-REM time points were extracted via a threshold at the trough between the peaks in the broadband PC1 histogram. Among the remaining time points, REM was recognized by finding time points with both EMG values below the bimodal dip in that metric and theta in the upper mode of the distribution. Waking was defined as 7 min or longer arousals. After automated brain state scoring, all states were manually reviewed by the experimenter and minor corrections were made when discrepancies between automated scoring and user assessment occurred (Watson et al., 2016).

##### Detection of UP and DOWN states

Slow waves were detected using the coincidence of a two-stage threshold crossing in two signals: a drop in high gamma power (100-400 Hz, representative of spiking) and a peak in the delta-band filtered signal (0.5-8 Hz) (Levenstein et al., 2018). The gamma power signal was smoothed using a sliding 80-ms window, and locally normalized to account for non-stationaries in the data. Two thresholds were used for event detection in each LFP-derived signal: a “peak threshold” and a “window threshold.” Time epochs in which the delta-filtered signal crossed the peak threshold were taken as putative slow wave events, with start and end times at the nearest crossing of the window threshold. To determine the delta threshold, all peaks in the delta-filtered signal greater than 0.25 standard deviations were detected as candidate delta peaks and binned by peak magnitude. The peri-event time histogram (PETH) for spikes from all cells was calculated around delta peaks in each magnitude bin and normalized by the mean rate in all bins. The smallest magnitude bin at which spiking (i.e., the PETH at time = 0) was lower than a set rate threshold was taken to be the peak threshold. The window threshold was set to the average delta value at which the rate crosses this threshold in all peak magnitude bins. The gamma power threshold was calculated similarly but using a drop below a gamma power magnitude instead of peaks above a delta magnitude. Once the thresholds were calculated, candidate events were then detected in the delta and gamma power signals, and further limited to a minimum duration of 40 ms. Slow wave events were then taken to be overlapping intervals of both the gamma and delta events.

##### Depth normalization in V1

To calculate standardized depth coordinates across animals, we aligned depth profiles using electrophysiological landmarks. These included the largest amplitude peak of the depth profile of high-frequency LFP power (500 Hz - 5 kHz) corresponding to mid-layer 5 and four prominent sinks and sources from the averaged DOWN-UP CSD maps for each animal (see Figure 1). Depth profiles from the different animals were aligned according to these landmarks and the distance between each of them was “warped” (either extended or stretched) accordingly. Linear interpolation of inter-electrode distance was performed to obtain distance in μm. Estimated pia surface was 80 μm above landmark *a*.

##### Independent Component Analysis of LFPs

To separate the different sources that contribute to the LFP mixed signal, we employed independent component analysis (ICA) analysis as has been described and validated previously for hippocampal recordings (Fernández-Ruiz et al., 2012, Schomburg et al., 2014, Fernández-Ruiz et al., 2017). ICA is a blind source-separation technique (Comon, 1994) that can isolate spatially segregated stable patterns of activity in a mixed signal recorded with an array of sensors. Applied to linear profiles of LFP it can separate physiologically meaningful sources. Here, we applied ICA to spatially contiguous LFP channels after filtering in the gamma band (30 – 100 Hz). Prior to application of the ICA algorithm, we performed a principal component analysis (PCA) reduction and maintained only the first ten PCs for subsequent ICA decomposition. The ICA algorithm (*runinca;* Bell and Sejnowski, 1995) takes a time series of data with dimension equal to the number of recording sites, and returns a time series of the same dimensionality, but rotated so that each dimension represents a different IC. The inverse of the mixing matrix that transforms the LFP data into the ICs gives the channel weight of each component that is captured for each electrode. When projected to the anatomical location of the electrodes, this corresponds to the spatial voltage loadings of each IC (Fernández-Ruiz et al., 2012). We ranked the components by the amount of variance they explain in the original data (relative power).

##### Gamma coherence and gradient descent clustering

Using coherence as similarity measure, an interaction-energy based clustering was implemented as previously done for hippocampal LFP^7^. Every site served as a reference against all the other referred sites. The resulting values were clustered using a gradient-descent algorithm, so that each site was merged with that cluster for which the resulting coherence gain after merging was the largest. Starting from random initial assignments, the clustering algorithm formed stable but fewer clusters corresponding to a local energy minimum. Energy of cluster A is defined as:

$$E^{A}=\;\frac{-1}{N^{A}}\;\sum_{i,j\in A}C_{ij}$$

Where, C_ij_ is the coherence between *i*th and *j*th sites and NA is the number of recording sites in cluster A. The energy gap between two different assignments to cluster A and B of site *i* is:

$$\Delta E_{i}^{AB}=\;\frac{1}{N^{B}}\sum_{j\in B}C_{ij}-\frac{1}{N^{A}}\sum_{k\in A}C_{ik}$$

If the energy gap is positive, site *i* is moved into cluster B, otherwise it remains in cluster A. Since the method results only local minima of energy and stochastic components, such as the random initial condition and update order affects, clustering consistency was verified by repeating the process several times.

##### Spectral analysis, cross-frequency coupling, and spike-LFP coupling

To perform spectral analysis at a high resolution in time and frequency, the complex wavelet transform (CWT) of the LFP (or ICs) was calculated using complex Morlet wavelets (Torrence and Compo, 1998). Wavelets were calculated using a logarithmically spaced frequency vector in the band of interest. Phase-amplitude cross-frequency coupling for a given LFP recording was assessed using the modulation index measure (MI; Tort et al., 2008). Phase time-series were binned into phase intervals and the mean wavelet amplitude was calculated for each of them. The MI was obtained by measuring the divergence of the observed amplitude distribution from the uniform distribution. The statistical significance of the MI values (p value) was assessed by a surrogate analysis (n = 1000 surrogates) with random shifts between the phase and amplitude time series. Mean-subtracted spectral analysis was obtained by calculating the mean power or coherence across all V1 channels for each session and subtracting it from each channel value. For the presented plots, grand averages were calculated as the mean across all animals.

The phase-locking of spikes to LFP features at each frequency was measured for individual units using the wavelet phase from 30-100 Hz (20 logarithmically spaced wavelet scales) at the time of each spike^9^. Modulation indices were calculated using the mean resultant length of the phases, and significance was estimated using the Rayleigh test for non-uniformity (p < 0.05) using circular statistics. Preferred frequency of modulation was determined as the largest mean vector length of each significantly modulated neuron.

##### Classification of units using spike-LFP phase coupling

To classify V1 units, we took advantage of the layer-specificity of gamma oscillations (Figure 2). For each unit, we first calculated their spike-phase modulation by the 6 gamma ICs. Next, we used the matrix of spike-IC phase coupling of all V1 units (excitatory and inhibitory) against all six ICs to perform k-Means clustering. The ‘cityblock’ metric was used as distance measure. We reduced the dimensionality of the data with t-Distributed Stochastic Neighbor Embedding (t-SNE) method by optimizing the Kullback-Leibler divergence of distributions between the original space and the embedded space, computing the Mahalanobis distance. We found the optimal number of clusters in the data to be eight by employing the silhouette criterion.

##### Calculation of unit burstiness

Burstiness was determined by calculating the average number of spikes in the 1.5–13.5 ms bins of the spike autocorrelogram divided by the average number of spikes in the 200–300 ms bins.

### Quantification and Statistical Analysis

#### Statistical analysis

All statistical analyses were performed with standard MATLAB functions. No specific analysis to estimate minimal population sample were used, but the number of animals, trials, and recorded cells were larger or similar to those employed in previous works. Unless otherwise noted, for all tests, non-parametric two-tailed Wilcoxon rank-sum (equivalent to Mann-Whitney U-test), Wilcoxon signed-rank or Kruskal-Wallis one-way analysis of variance were used. For multiple comparisons, Tukey’s honesty post hoc test was employed. Boxplots represent median and 25^th^/75^th^ percentiles and their whiskers the data range. In some of the plots outlier values were not shown but they were always included in the statistical analysis.

### Data and Software Availability

LFP and spike data have been deposited in buzsakilab.com and are freely available for further analyses.

## Supplemental Information

Document S1. Figures S1–S8

Document S2. Article plus Supplemental Information
