# Supplementary material for: Layer-Specific Physiological Features and Interlaminar Interactions in the Primary Visual Cortex of the Mouse
Source: Neuron. 2019 Feb 6;101(3):500–513.e5. doi: 10.1016/j.neuron.2018.12.009 (PMC6367010; doi:10.1016/j.neuron.2018.12.009)
Supplement: Document S1. Figures S1–S8 [file mmc1.pdf]

Neuron, Volume *101*

Supplemental Information

Layer-Specific Physiological Features and Interlaminar Interactions in the Primary Visual Cortex of the Mouse

Yuta Senzai, Antonio Fernandez-Ruiz, and György Buzsáki

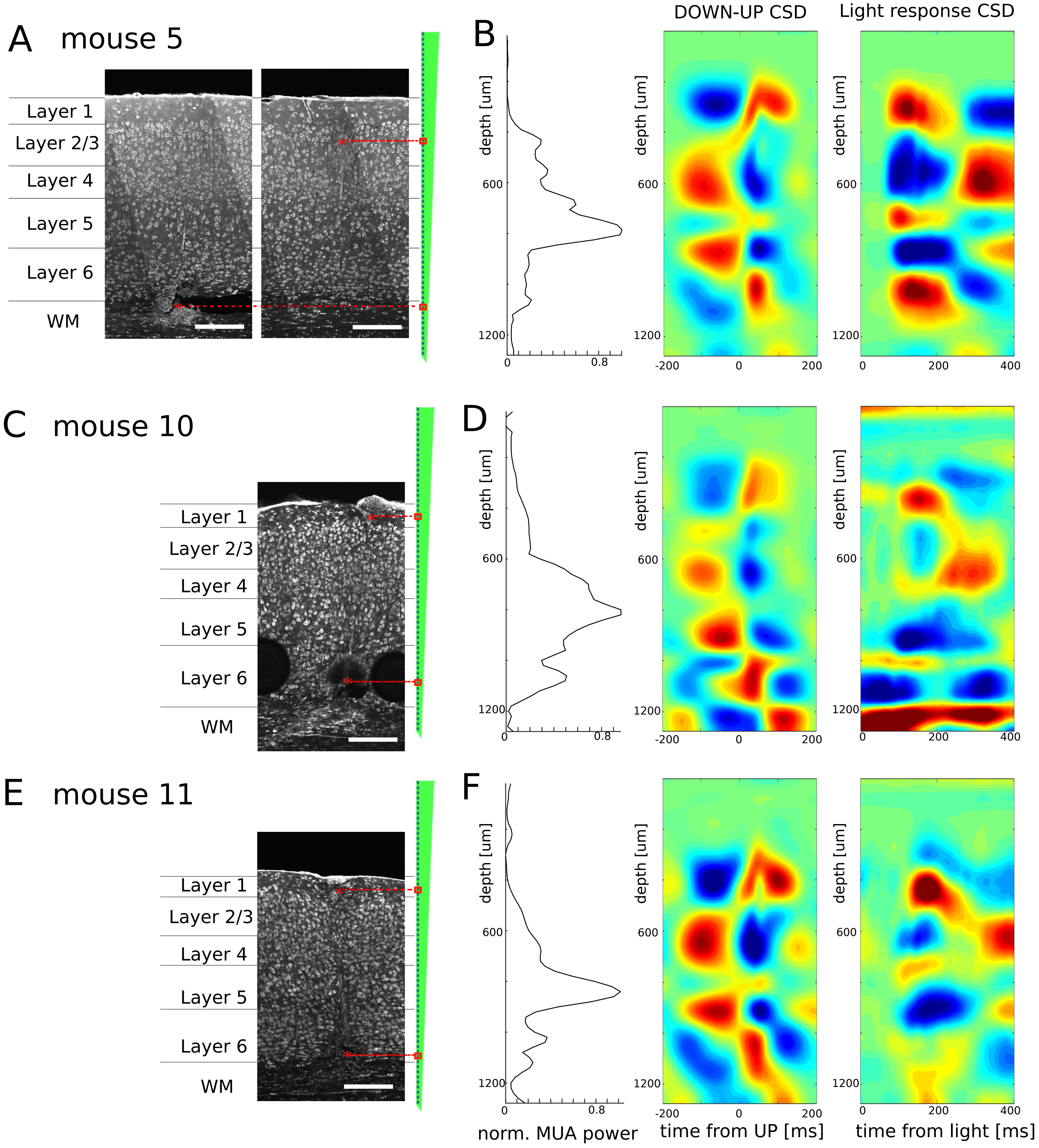

A
mouse 5
B
DOWN-UP CSD
Light response CSD
Layer 1
Layer 2/3
Layer 4
Layer 5
Layer 6
WM
C
mouse 10
D
E
mouse 11
F
depth [um]
600
1200
0
0.8
-200
200
400
norm. MUA power
time from UP [ms]
time from light [ms]

Figure S1: **Histological verification of probe location and physiological landmarks, related to Figure 1.**

Additional examples of histological verification of probe location along V1 cellular layers (**A, C, E**). Small electrolytical lesions were performed with the two electrodes highlighted on the silicon probe sketch. **B, D, F**) Multi-unit (MUA) spectral power (500 Hz – 5 kHz) distribution as a function of depth and average CSD map for DOWN-UP transitions of non-REM sleep and for visual stimulation when available. Horizontal dashed lines indicate subjective layer borders estimated from the histological sections.

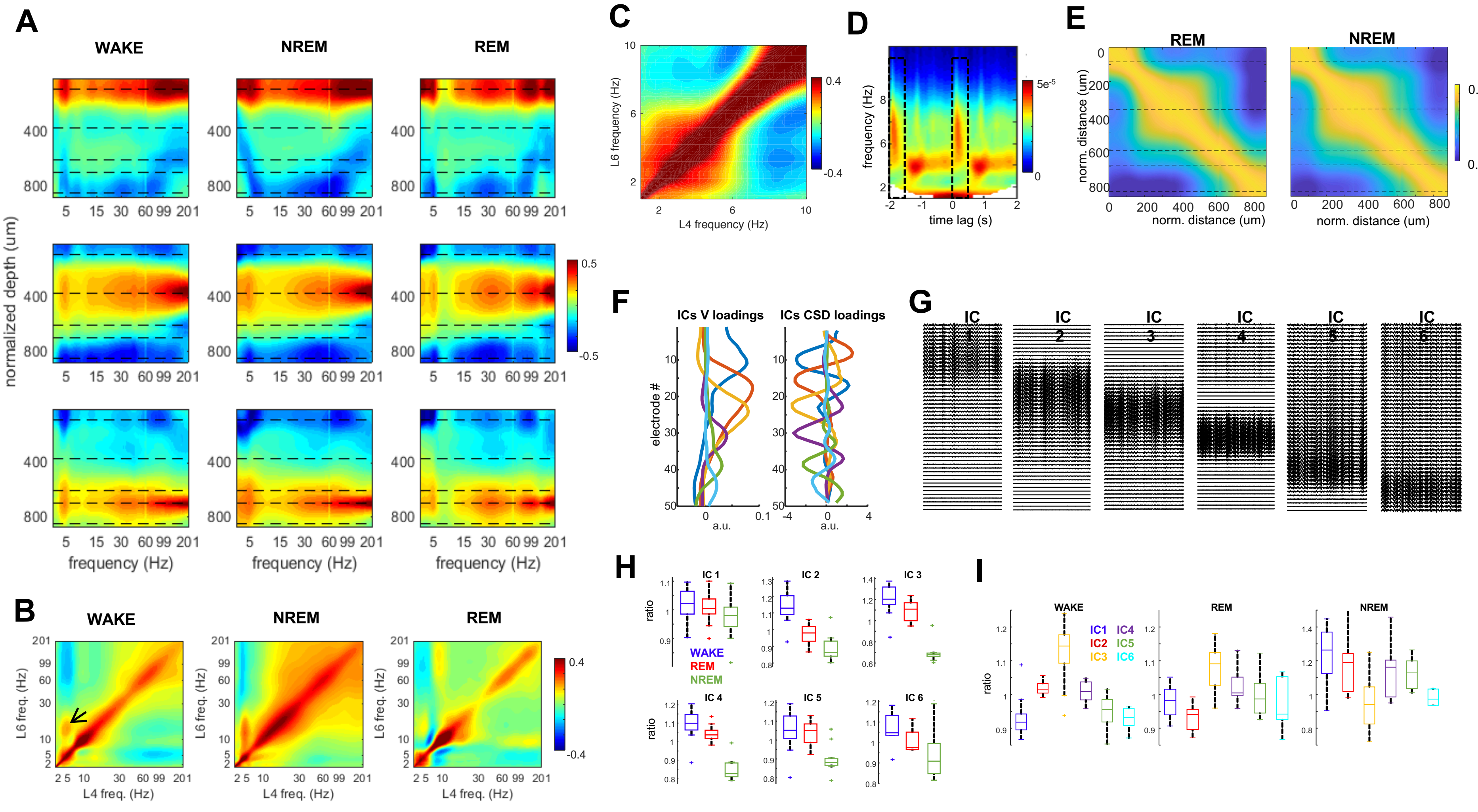
A
WAKE
NREM
REM
normalized depth (um)
frequency (Hz)
B
L6 freq. (Hz)
L4 freq. (Hz)
C
L6 frequency (Hz)
L4 frequency (Hz)
D
frequency (Hz)
time lag (s)
E
norm. distance (um)
F
ICs V loadings
ICs CSD loadings
electrode #
a.u.
G
IC 1
IC 2
IC 3
IC 4
IC 5
IC 6
H
ratio
WAKE
REM
NREM
I
IC1 IC4
IC2 IC5
IC3 IC6

Figure S2: **Decomposition of cortical layers by gamma LFPs, related to Figure 2.**

A) Averaged mean-subtracted coherence spectra during waking, non-REM and REM, referenced to a recording site in the superficial (top), layer 4 (middle) or deep layer (bottom) (n = 19 mice; colorbar: mean-subtracted coherence value). **B**) Averaged cross-frequency power-power comodulation in different behavioral states between LFP in layers 4 and 6 (colorbar: power correlation). Note high comodulation between the 3-6 Hz band and 15-25 Hz band (arrow). **C**) Averaged cross-frequency power-power comodulogram showing the strong anti-correlation between theta and 3-6 Hz bands (colorbar: power correlation). **D**) Averaged wavelet spectrogram after presentation of visual stimuli (400 ms duration, dashed lines; colorbar: wavelet amplitude). Note the appearance of a 4 Hz power bump after stimulus offset. **E**) Averaged pair-wise gamma (30-100 Hz) coherence matrices for all LFP channels in normalized coordinates in non-REM and REM (n = 19; colorbar: coherence). Note similar gamma matrices in all brain states (compare with Figure 4A). **F**). Example of ICA decomposition of gamma band LFPs into 6 main components (ICs) for one session. Color lines represent voltage (V) and CSD depth profiles for each component. **G**) Reconstructed LFPs for each component show laminar stratification of gamma oscillations. **H**) Variance of each component relative to the mean across brain states for all animals. Note higher relative power during waking. **I**) Variance of each component during waking, REM and non-REM relative to the mean across ICs for all animals. Note that each brain state is characterized by a different relative contribution of gamma-ICs.

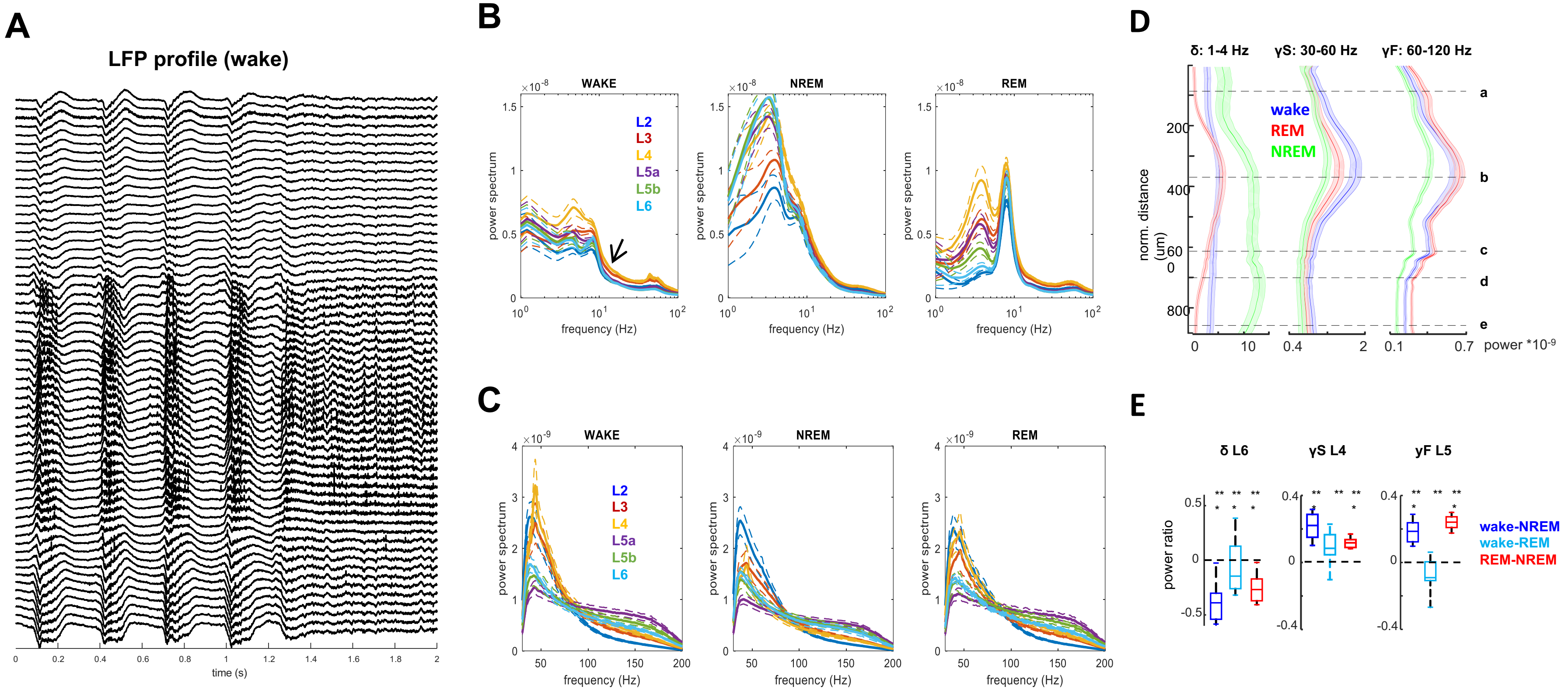

A
LFP profile (wake)
time (s)
B
WAKE
NREM
REM
L2
L3
L4
L5a
L5b
L6
power spectrum
frequency (Hz)
C
WAKE
NREM
REM
power spectrum
frequency (Hz)
D
δ: 1-4 Hz
γS: 30-60 Hz
γF: 60-120 Hz
wake
REM
NREM
norm. distance (um)
a
b
c
d
e
power *10-9
E
δ L6
γS L4
γF L5
power ratio
wake-NREM
wake-REM
REM-NREM

Figure S3: **Behavior-dependence of spectral patterns and cortical layer-identification by LFP criteria, related to Figure 3.**

A) Example LFP depth profile during wake showing an episode of 3-6 Hz oscillations. **B**) Averaged LFP power spectra in different layers and brain states (mean ± SEM, log scale; n = 19 mice). Layers were determined by LFP criteria of Figure 3. The peak at 6-9 Hz in wake and REM is volume-conducted theta oscillation from the hippocampus. Note stronger slow oscillations during NREM and increased gamma power in superficial layers in waking (arrow). Note log x scale. **C**) Averaged power spectra in different layers (ICs) and brain states (linear scale) to emphasize the different properties of low frequency gamma 30-60 Hz) and high frequency gamma (>100 Hz). Note the sharp slow gamma peak in L4 IC (yellow). **D**) Depth profiles of brain state-dependent spectral power in different frequency bands (mean ± SEM, n =19). Note larger gamma power in the superficial layers and its increase during waking. Note also larger power of delta band in deep layers and during non-REM. Dashed lines indicate landmarks as in Figure 1. **E**) Brain state-dependent shift of power in different frequency bands. */**/*** $p < 0.05/0.01/0.001$, signrank test.

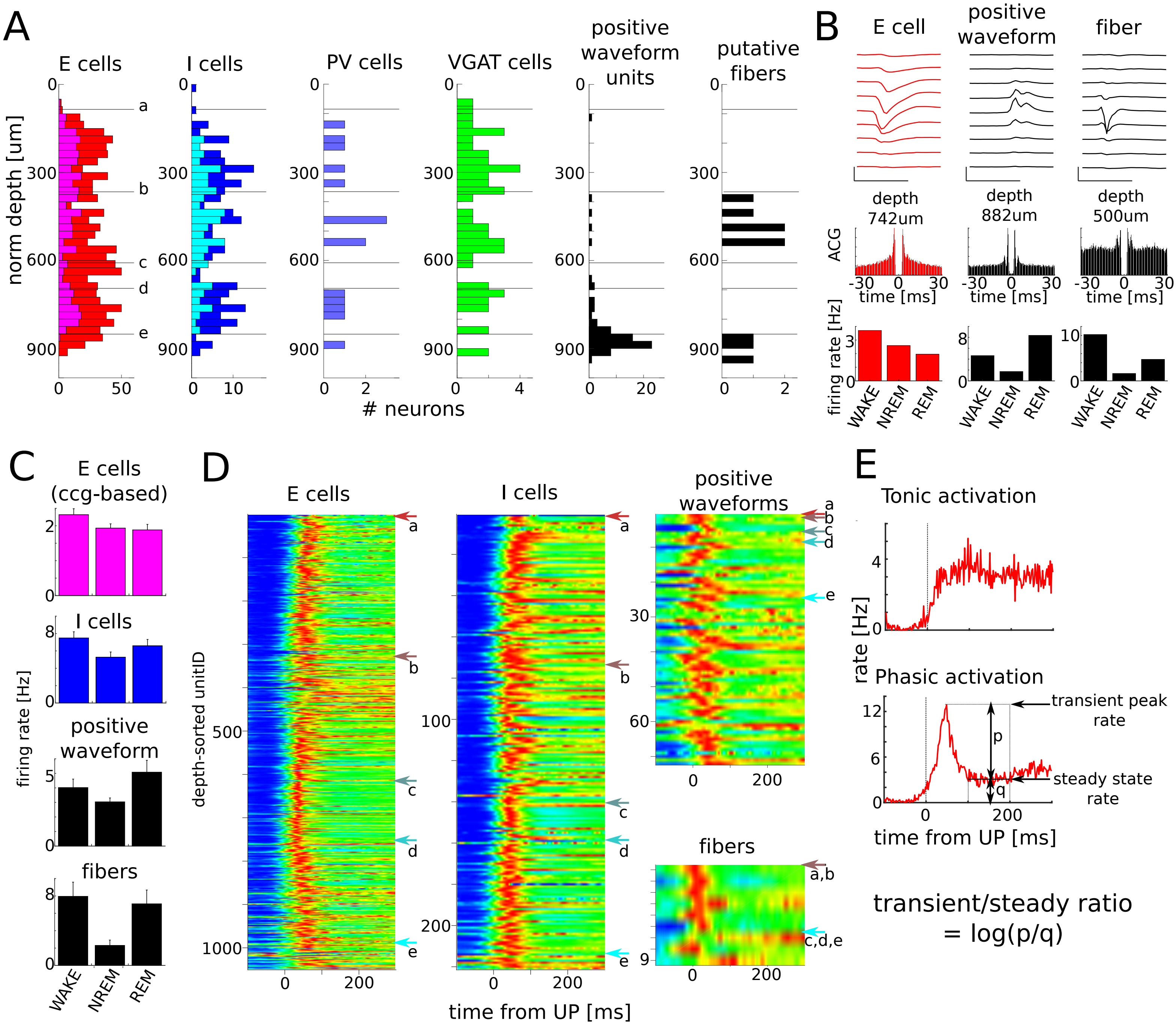
A
E cells
I cells
PV cells
VGAT cells
positive waveform units
putative fibers
norm depth [um]
neurons
B
E cell
positive waveform
fiber
depth 742um
depth 882um
depth 500um
ACG
time [ms]
firing rate [Hz]
WAKE
NREM
REM
C
E cells (ccg-based)
I cells
positive waveform
fibers
firing rate [Hz]
D
E cells
I cells
positive waveforms
fibers
depth-sorted unitID
time from UP [ms]
E
Tonic activation
Phasic activation
rate [Hz]
transient peak rate
steady state rate
time from UP [ms]
transient/steady ratio = log(p/q)

Figure S4: **Depth distribution of different neuron types, related to Figures 4 and 6. A**) Normalized depth distribution of putative E (red) and I (blue) neurons. Magenta and light blue colors refer to physiologically identified subgroups based on monosynaptic latency cross-correlation peaks or troughs (Figure 2C, D). Also shown are subgroups of optogenetically identified PV-expressing and GABA-expressing (VGAT) neurons, positive waveform cells and putative short duration fiber potentials. **B**) Examples of action potentials and autocorrelograms of an E cell, positive waveform cell and a putative fiber potential. **C**) Median firing rates for different neuron types during wake, non-REM and REM states. Arrows indicate normalized landmarks. **D**) Normalized firing rate histograms as a function of recorded depth for E and I cells and positive waveform neurons during DOWN-UP transition of slow oscillations of non-REM. **D**) Examples of sustained firing neuron (top) and transient-steady neuron (bottom). The calculation of phasic-steady index is illustrated.

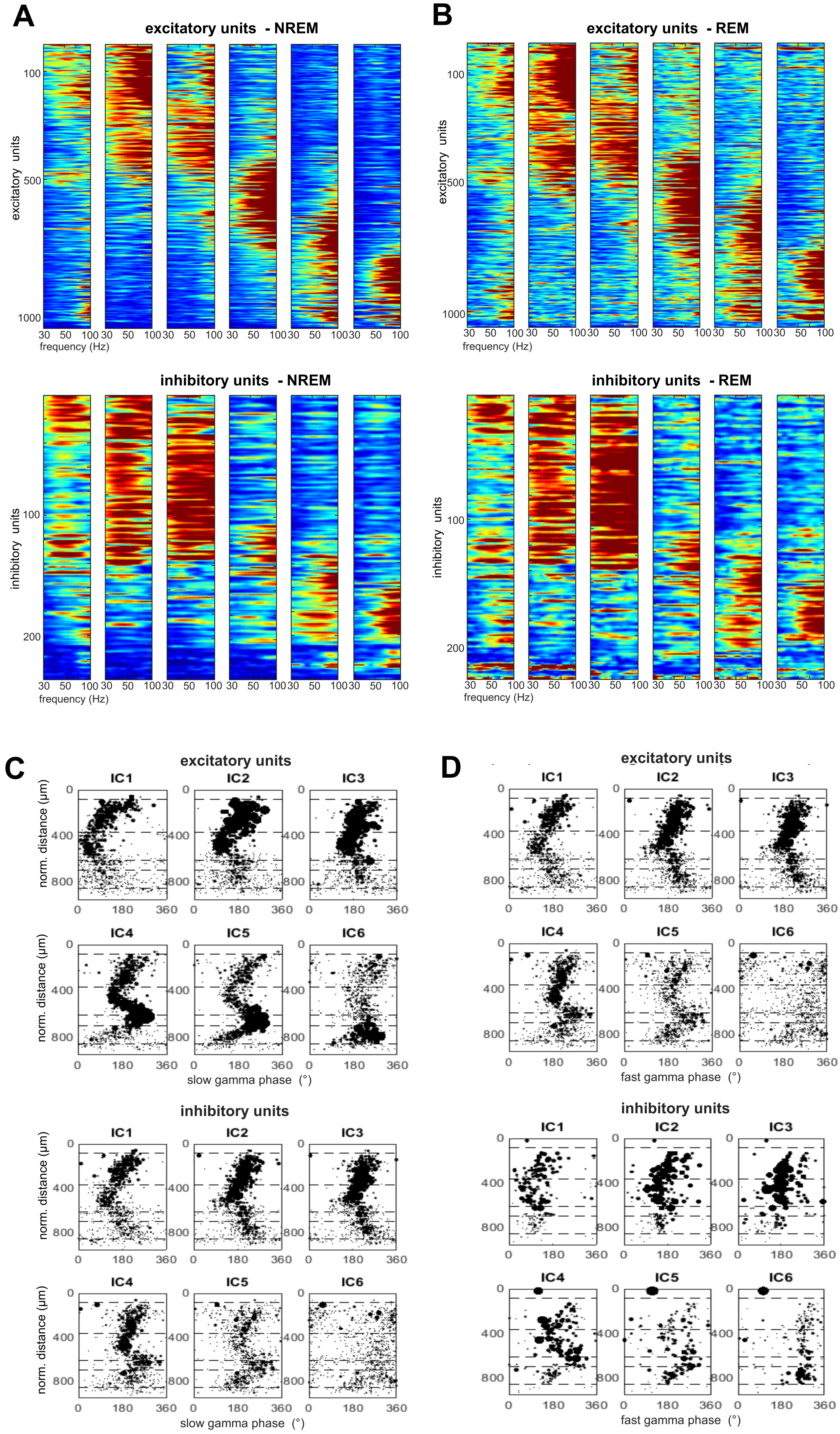
A
excitatory units - NREM
excitatory units
frequency (Hz)
inhibitory units - NREM
inhibitory units
B
excitatory units - REM
inhibitory units - REM
C
excitatory units
IC1
IC2
IC3
IC4
IC5
IC6
norm. distance (µm)
slow gamma phase (°)
inhibitory units
D
fast gamma phase (°)

Figure S5: Identification of cell body layers by spike-LFP criteria, related to Figure 5.

A) Spike-gamma IC coherence for each principle cell (top) and interneuron (bottom) during non-REM and REM (**B**). In each panel the reference signal corresponds to one of the 6 ICs. Only units with significant modulation ($P < 0.05$; Rayleigh test) with the gamma band are displayed. Compare with Figure 5A (waking).**C**) Unit versus depth location and preferred phase of slow (30-60 Hz) or fast gamma (**D**; 60-100 Hz). In each panel the reference signal corresponds to one of the 6 ICs. Top, principal cells; bottom: interneurons. Size of dots is proportional to the magnitude of unit-IC coupling (mean vector length).

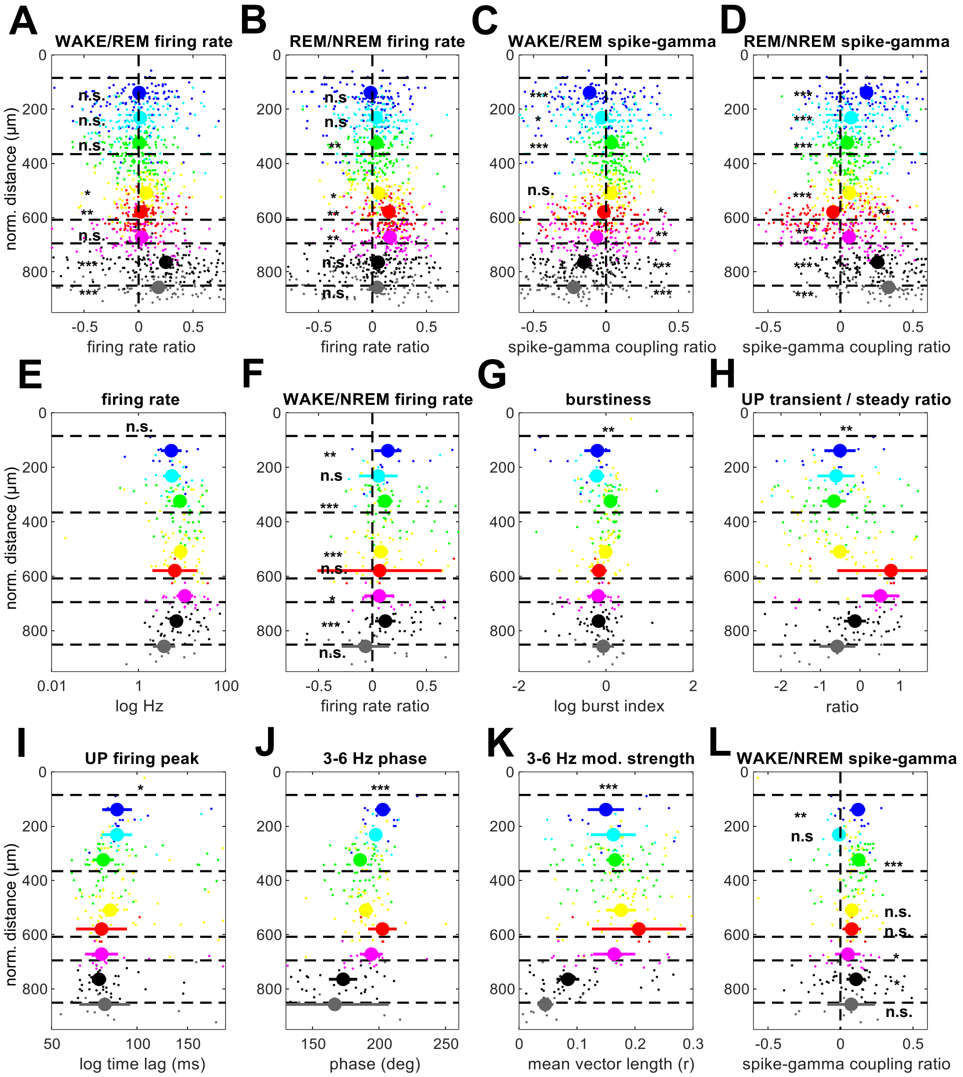

A
WAKE/REM firing rate
B
REM/NREM firing rate
C
WAKE/REM spike-gamma
D
REM/NREM spike-gamma
E
firing rate
F
WAKE/NREM firing rate
G
burstiness
H
UP transient / steady ratio
I
UP firing peak
J
3-6 Hz phase
K
3-6 Hz mod. strength
L
WAKE/NREM spike-gamma
norm. distance (µm)
firing rate ratio
spike-gamma coupling ratio
log Hz
log burst index
ratio
log time lag (ms)
phase (deg)
mean vector length (r)
n.s.

Figure S6: Physiological properties of neurons in different layers, related to Figure 6.

A to D) Comparisons of state-dependent firing rates and spike-LFP gamma phase modulation across brain states (WAKE/REM; REM/NREM). */**/*** $p < 0.05/0.01/0.001$, ranksum or Kruskall-Wallis test. **E to L**) Physiological properties of putative interneurons in different cortical layers. Compare the panels to Figure 6 for putative excitatory cells. Colors correspond to the 8 clusters of Figure 5C

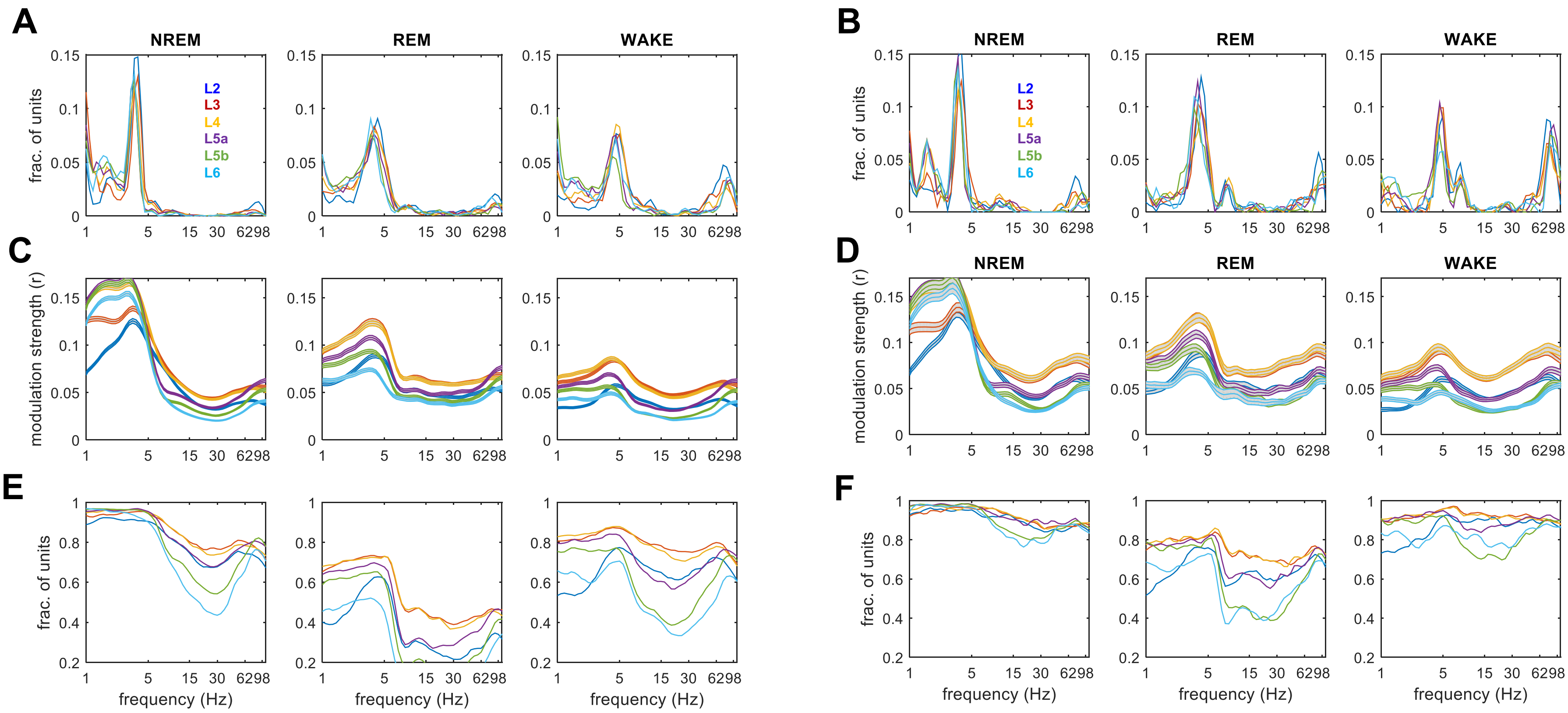
A
B
C
D
E
F
NREM
REM
WAKE
L2
L3
L4
L5a
L5b
L6
frac. of units
modulation strength (r)
frequency (Hz)

Figure S7: Modulation of V1 units by slow, 3-6 Hz and gamma oscillations, related to Figures 3 and 6.

A, B) Distribution of preferred frequencies for significantly modulated excitatory (A) and inhibitory (B) cells. Recordings from different layers are color-coded. Note large peak in 3-6 Hz and in the gamma band during wake, indicating that the majority of neurons are coupled to these frequency bands. **C, D**) Spike – LFP phase modulation strength related at different frequencies and in different layers layers (mean ± SEM). Note strongest phase-modulation of units by the 3–6 Hz oscillation and slow oscillation (< 2 Hz). **E, F**) Fraction of significantly modulated excitatory and inhibitory units. Note that the largest fraction of LFP-modulated neurons resides in superficial layers.

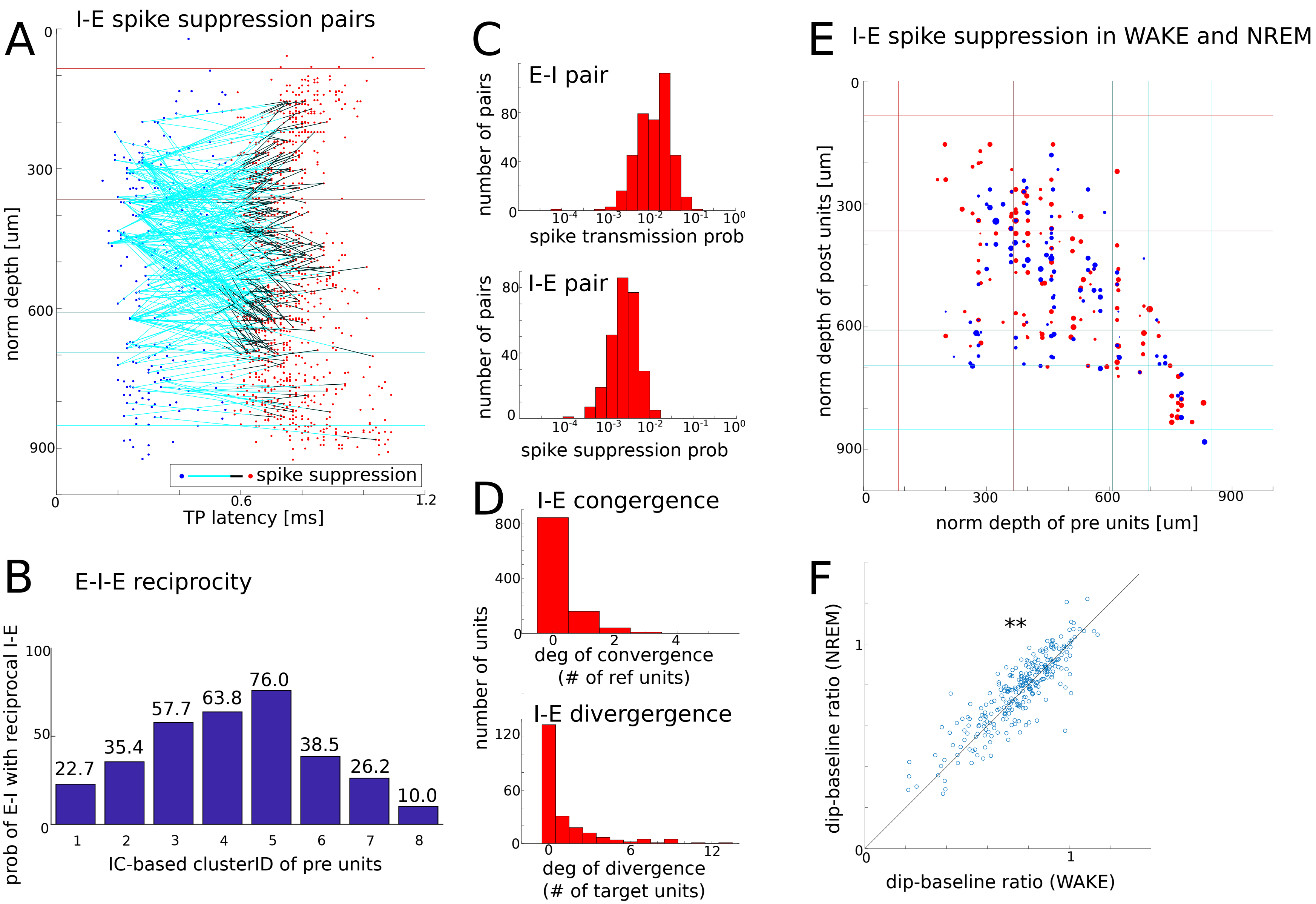

A
I-E spike suppression pairs
norm depth [um]
TP latency [ms]
spike suppression
B
E-I-E reciprocity
prob of E-I with reciprocal I-E
IC-based clusterID of pre units
22.7
35.4
57.7
63.8
76.0
38.5
26.2
10.0
C
E-I pair
number of pairs
spike transmission prob
I-E pair
spike suppression prob
D
I-E congergence
number of units
deg of convergence
(# of ref units)
I-E divergergence
deg of divergence
(# of target units)
E
I-E spike suppression in WAKE and NREM
norm depth of post units [um]
norm depth of pre units [um]
F
dip-baseline ratio (NREM)
dip-baseline ratio (WAKE)
**

Figure S8: I-E spike transmission probability changes across brain states, related to Figure 7.

A) I-E connections. Light blue lines with black ends indicate putative monosynaptic pairs from I to E neurons. **B**) Probability of reciprocal I-E connections of the E-I connected pairs. Percentages of the reciprocal connections in each unit cluster group are shown above the graphs. Note largest fraction of reciprocal excitatory-inhjbitory connections among pairs of clusters 4, 5 and 5 (layer 5). **C**) Distribution of E-I and E-I connection strength for all pairs. Note normal distribution on a log scale. D) E) Distribution of the I-E divergence and convergence for all pairs. E) I-E spike transmission probability change across waking and non-REM states. The size of the dots indicates the difference of spike transmission probability between states. Red, stronger suppression during wake; blue, stronger suppression during non-REM. F) I-E spike suppression during waking and non-REM. ** $P < 0.01$ (signrank test).
